# Progressive correction of auditory hair cell orientation in the absence of core planar cell polarity and GPR156 signaling

**DOI:** 10.1242/dev.205332

**Published:** 2026-05-14

**Authors:** Amandine Jarysta, Justin Nemelka, Muthumeena Ramanathan, Ellison J. Goodrich, Brianna L. Vonderhaar, Sungjin Park, Cesare Orlandi, Michael R. Deans, Basile Tarchini

**Affiliations:** ^1^ The Jackson Laboratory, Bar Harbor, ME 04609, USA; ^2^ Department of Neurobiology, Spencer Fox Eccles School of Medicine at the University of Utah, Salt Lake City, UT 84112, USA; ^3^ Graduate School of Biomedical Science and Engineering, University of Maine, Orono, ME 04469, USA; ^4^ Department of Cell Biology, Emory University School of Medicine, Atlanta, GA 30322, USA; ^5^ Department of Pharmacology and Physiology, University of Rochester Medical Center, Rochester, NY 14642, USA; ^6^ Tufts University School of Medicine, Boston, MA 02111, USA

**Keywords:** Inner ear, Cochlea, Sensory hair cell, Planar cell polarity

## Abstract

Planar cell polarity (PCP) proteins, including Van Gogh-like and frizzled, orient sensory hair cells in the inner ear. Because most PCP mouse models die at birth, orientation defects have been studied mainly at fetal stages. Using viable conditional mutants lacking both *Vangl1* and *Vangl2*, or *Fzd3* and *Fzd6*, we show that misoriented auditory hair cells undergo extensive postnatal reorientation, largely correcting defects over time. These findings extend previous observations in *Vangl2* single conditional mutants and demonstrate that correction does not require redundant core PCP partners. GPR156, a receptor that reverses how PCP signaling is interpreted in some hair cell types, is also essential for orientation but *Gpr156* mutants fail to correct defects. We therefore tested its role in core PCP mutant recovery. GPR156 binds and depends on VANGL proteins, yet inhibiting GPR156 signaling facilitates rather than prevents correction in *Vangl* and *Fzd* mutants. Thus, GPR156 collaborates with core PCP components to establish hair cell orientation but is dispensable – and even inhibitory – for postnatal realignment. These findings reveal extensive plasticity in hair cell orientation and identify GPR156 as a context-dependent partner of core PCP proteins.

## Teaser

**Summary:** Misoriented mouse auditory hair cells can spontaneously correct their orientation after birth, revealing unexpected plasticity and a partnership between core planar cell polarity proteins and the GPR156 receptor.

## INTRODUCTION

Sensory hair cells (HCs) in the cochlea detect sound vibrations through a specialized apical structure known as the stereociliary bundle, or hair bundle. This bundle comprises highly organized and stable membrane protrusions supported by a F-actin backbone, the stereocilia. A defining feature of hair bundles is their asymmetric architecture, where stereocilia are arranged in rows of graded heights, which imparts directionality to the HC response when the hair bundle is mechanically deflected. Multiple classes of cell polarity proteins act autonomously within HCs to establish a planar-asymmetric cytoskeleton at the apical surface during development (Kindt and Tarchini, 2025; Tarchini and Lu, 2019). However, HCs do not construct a directional sensor in isolation. To effectively detect sound waves traveling along the cochlear duct, HCs must also be correctly aligned within the auditory epithelium, with their orientation coordinated across neighboring cells. This organ-level organization is controlled in part by highly conserved apical junction complexes formed by core planar cell polarity (PCP) proteins. In many systems, a Van Gogh-like based complex and a frizzled-based complex segregate to opposite junctions in the cell and are selectively paired across adjacent cells via homotypic dimerization of CELSR1, a component common to both complexes (Aw and Devenport, 2017). In the auditory epithelium, core PCP signaling regulates adhesion between each HC and its direct supporting cell neighbors, enabling the local propagation of polarity information across the epithelium.

As a result, mouse core PCP mutants, including *Vangl1; Vangl2* and *Fzd3; Fzd6* double mutants, exhibit HC misorientation, with variability in the affected HC types and the severity of defects, depending on the specific core PCP protein and mutant allele involved (Montcouquiol et al., 2006; Wang et al., 2006b; Yin et al., 2012). Misorientation is characterized by a rotation of the entire apical cytoskeleton, including the graded hair bundle, the off-center basal body and its associated primary cilium, the kinocilium, among other structures. Notably, although these structures are misaligned relative to the epithelium, they typically remain internally coherent and properly patterned. Separately, core PCP proteins also regulate cell junction remodeling processes such as convergent extension (Sutherland et al., 2020). Consequently, neural tube closure is often compromised in constitutive core PCP mutants, resulting in perinatal lethality. One study circumvented this limitation by conditionally inactivating *Vangl2* in the inner ear, thereby preserving neural tube closure and allowing mutant mice to survive postnatally (Copley et al., 2013). Unexpectedly, a progressive correction of auditory HC orientation was observed during postnatal development. Since other core PCP proteins appeared depleted in absence of VANGL2, reorientation was proposed to rely on a distinct mechanism that remained to be identified.

More recently, the orphan G protein-coupled receptor (GPCR) GPR156 previously unrelated to the core PCP machinery was identified as essential for proper HC orientation. Specifically, studies have shown that GPR156 signals via inhibitory G proteins (GNAI, or Gαi) to reverse HC orientation in sensory regions of the inner ear that express the transcription factor EMX2 (Jia et al., 2023; Kindt et al., 2021; Ono et al., 2024; Watkins and Orlandi, 2021). Like core PCP proteins, GPR156 is asymmetrically enriched at the apical junction, but its expression is restricted to HCs. While the distribution of core PCP proteins does not appear to depend on GPR156, GPR156 is abnormally enriched in semi-dominant *Vangl2 Looptail* mutant HCs (Kindt et al., 2021). GPR156 appears to reverse how developing HCs interpret distinct, invariant core PCP cues on opposite sides of the apex as they are about to break symmetry, effectively reversing the resulting HC orientation. Although all cochlear HCs show the same polarized GPR156 enrichment, only the first two rows of outer HCs (OHCs; OHC1-2) are severely affected in *Gpr156* constitutive mutants, exhibiting an inverted orientation. Intriguingly, this phenotype resembles that of two core PCP mutant models: in constitutive *Fzd3; Fzd6* mutants, inner HCs (IHCs) are generally inverted, while OHCs are only mildly misoriented (Wang et al., 2006b). In *Vangl2* mutants, misorientation is most severe in OHC3 and also trends towards inversion (Yin et al., 2012). These results are somewhat unexpected because core PCP proteins are co-dependent: loss of one component typically disrupts the entire machinery (Montcouquiol et al., 2006; Yin et al., 2012), which would be expected to lead to randomized, rather than inverted, HC orientation. Finally, unlike in *Vangl2* mutants, HCs do not correct their orientation in *Gpr156* mutants (Kindt et al., 2021).

Functionally, both conditional *Vangl2* mutants (Copley et al., 2013) and constitutive *Gpr156* mutants (Kindt et al., 2021) have hearing impairment. In *Vangl2* mutants, residual OHC3 misorientation at the cochlear apex resulting from incomplete correction may contribute. In contrast, the severe and persistent misorientation of OHC1-2 is probably a root cause in *Gpr156* mutants. Indeed, conditional mutants where *Gpr156* inactivation is largely restricted to HCs also display hearing deficits (Jarysta et al., 2026). In addition, both *Vangl2* and *Gpr156* mutants show defects in supporting cell morphology, which are also likely to contribute to hearing loss.

In this study, we first establish that the postnatal correction of HC orientation initially reported in *Vangl2* mutants also occurs in *Vangl1; Vangl2* and *Fzd3; Fzd6* double mutants. Next, we show that HC reorientation does not rely on the overlying tectorial membrane and further demonstrate that this process can occur *ex vivo* in cochlear explants. Using explant culture, we investigate the relationship between core PCP proteins and GPR156, focusing on whether GPR156 influences the reorientation process. Our findings suggest that GPR156 interacts with core PCP components in HCs, and that inhibiting GPR156 signaling enhances reorientation in core PCP mutants, resulting in improved correction outcomes.

## RESULTS

### Postnatal correction of hair cell orientation in the absence of VANGL proteins

**Fig. 1.**
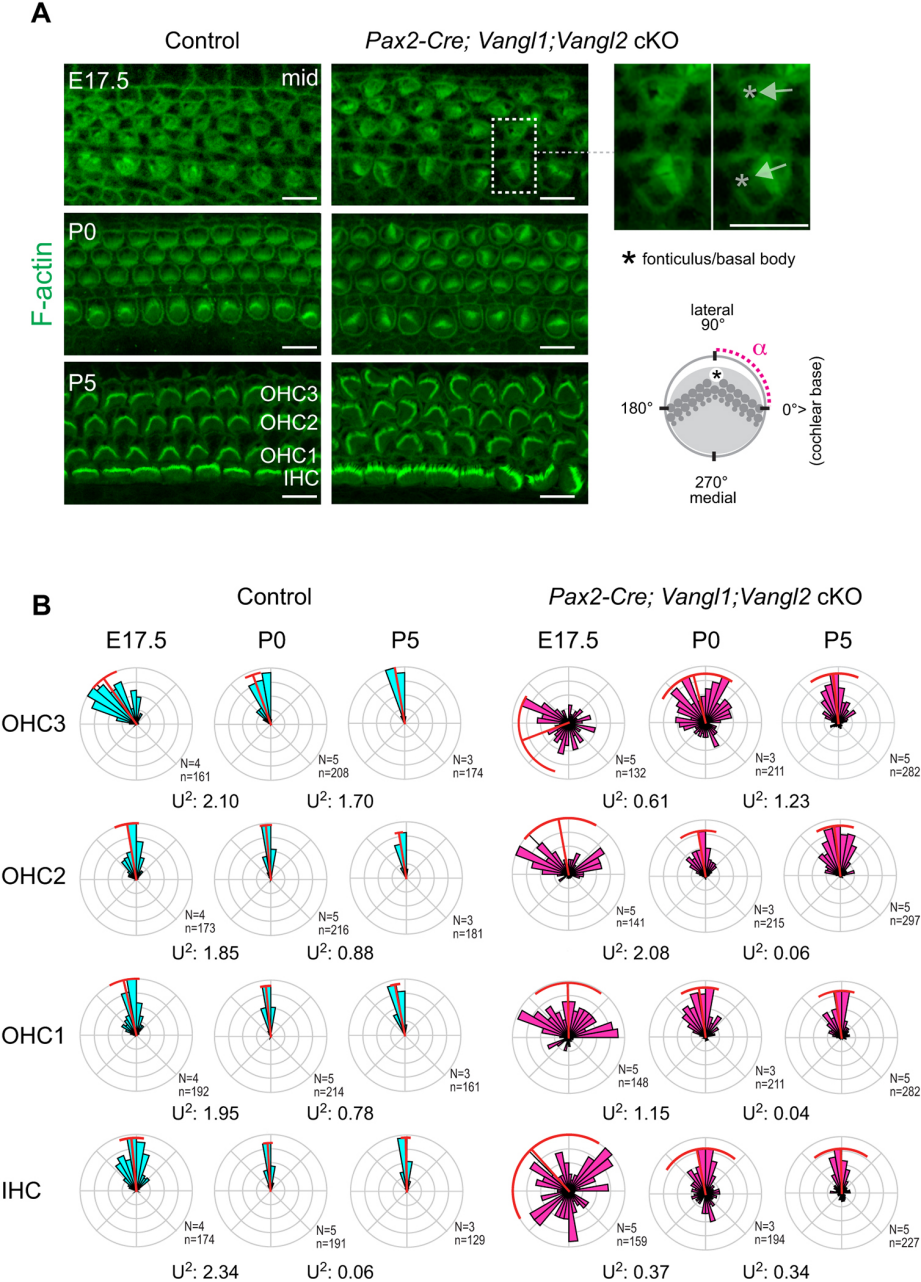
**Hair cell orientation in double conditional *Vangl1; Vangl2* mutants at E17.5, P0 and P5.** (A) Phalloidin (F-actin) labeling of the apical surface of the mouse auditory epithelium at E17.5, P0 and P5 at the mid-cochlear position. Area outlined is magnified on the right; arrows indicate HC orientation. The schematic represents the reference system where HC orientation (α) is measured based on the position of the hair bundle and fonticulus (asterisk) relative to the longitudinal axis of the cochlea. In this system, α=90° for lateral-oriented HCs and α=270° for inverted HCs. (B) Circular histograms representing HC orientation as a frequency distribution. Note how orientation in all HC types becomes more systematically lateral (90°) in *Vangl1; Vangl2* cKO (right) from E17.5 to P5, and thus more similar to corresponding control HCs (left). Red radial lines and arcs represent the circular mean and circular standard deviation, respectively. N and n represent the number of animals and HCs analyzed, respectively. Bin size is 10°. A Watson U^2^ test is used to compare angular distributions across stages. Control genotype is *Pax2-Cre; Vangl1^flox/+^*, *Vangl2^flox/+^*. *Vangl1; Vangl2* cKO genotype is *Pax2-Cre; Vangl1^del/flox^; Vangl2^del/flox^*. Scale bars: 10 μm.

Previous work established that a hallmark of core PCP dysfunction, cochlear HC misorientation, is largely corrected over time when *Vangl2* mutants are allowed to survive past birth using a conditional inactivation strategy (Copley et al., 2013). Although the polarized distribution of other core PCP proteins was disrupted in the absence of VANGL2, the close ortholog VANGL1 may play a compensatory role and explain phenotype erosion at postnatal stages. VANGL1 is asymmetrically localized at HC-support cell junctions in a pattern similar to VANGL2 (Belotti et al., 2012; Song et al., 2010), and *Vangl1*; *Vangl2* double mutants display more severe HC misorientation defects compared to *Vangl2* single mutants in the cochlea (Song et al., 2010) and in the vestibular system (Stoller et al., 2018). We thus generated a double conditional knockout of *Vangl1* and *Vangl2* using *Pax2-Cre* (*Pax2-Cre*; *Vangl1^del/flox^*; *Vangl2^del/flox^*, hereafter *Vangl1; Vangl2* cKO). *Pax2* is broadly expressed in the developing otic vesicle prior to HC specification but is absent from the neural tube, ensuring that *Pax2*-Cre-driven core PCP cKOs are viable (Ohyama and Groves, 2004). We imaged cochlear HCs at embryonic (E) day E17.5 and postnatal (P) days P0 and P5 at the mid-cochlear position (50% of cochlear length) (Fig. 1A). We then measured orientation by HC type based on the orientation of the hair bundle and the position of the fonticulus, an opening in the HC cuticular plate that is visible after phalloidin labeling and indicates the position of the basal body at the vertex of the hair bundle (Fig. 1A). Angles were measured in relation to the cochlear longitudinal axis so that a bundle correctly oriented towards the lateral edge will have a measured angle of 90° (α=90°) from the cochlear base. At E17.5, OHC1-3 and IHC all showed clear misorientation in *Vangl1; Vangl2* cKO, as previously reported (Song et al., 2010), including many inverted OHC3 and IHCs that pointed medially (Fig. 1B). By P0, the orientation profile of OHC1-2 was more similar to control animals, and the proportion of inverted OHC3 and IHCs had decreased in double mutants. At P5, nearly all mutant HCs showed a relatively normal, although imprecise, lateral orientation. HC orientation is thus being corrected over time in *Vangl1; Vangl2* cKO, demonstrating that VANGL1 is not responsible for the reorientation seen in *Vangl2* cKOs.

### Correction of hair cell orientation proceeds without tectorial membrane attachment

**Fig. 2.**
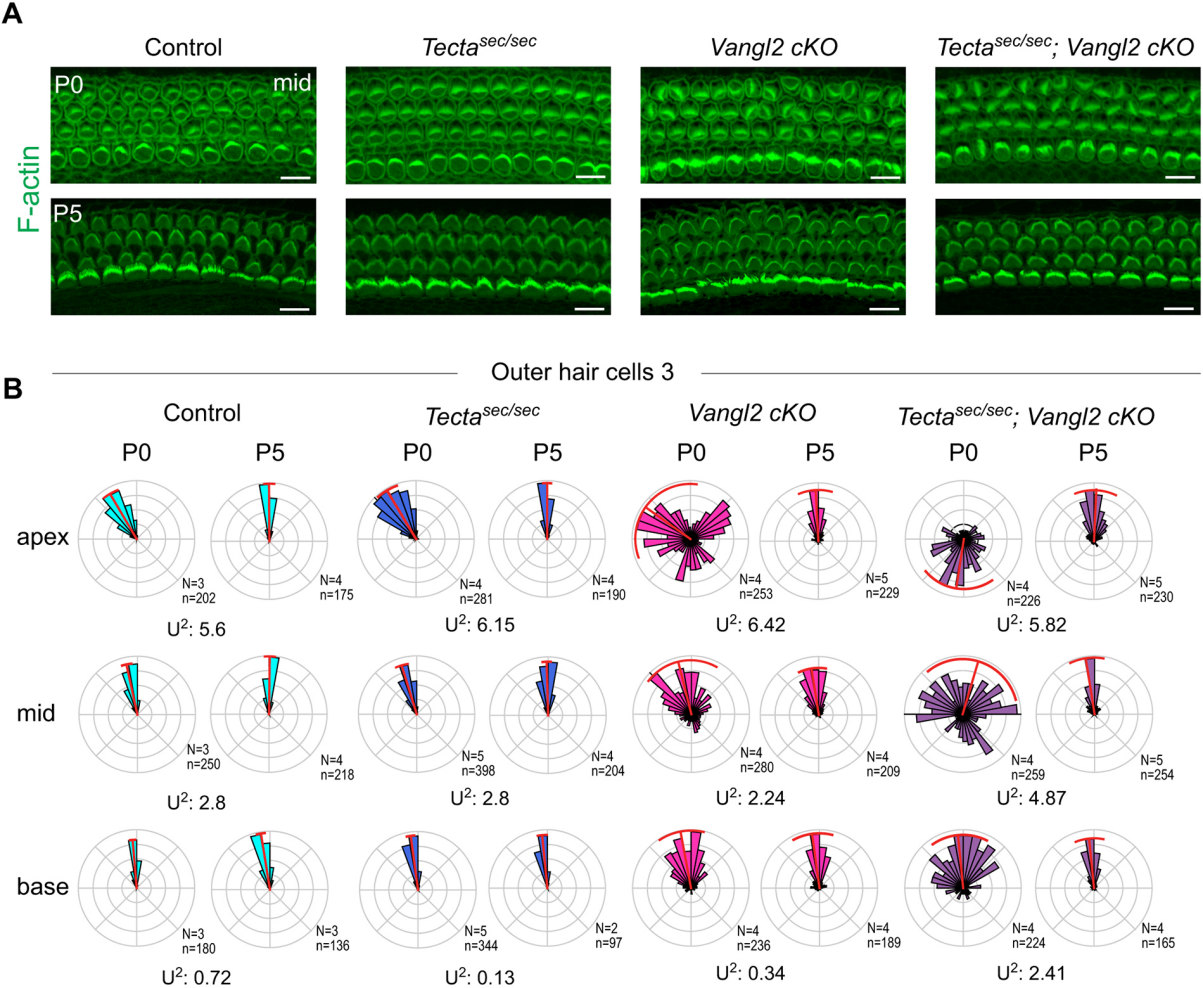
**Hair-cell orientation in conditional *Vangl2* mutants lacking tectorial membrane coverage at P0 and P5.** (A) Phalloidin (F-actin) labeling of the apical surface of the mouse auditory epithelium at P0 and P5 at the mid-cochlear position. (B) Circular histograms representing OHC3 orientation at the cochlear base, mid and apex positions. Note how OHC3, the most affected HC type in *Vangl2* cKO mutants, reorient to correct their orientation between P0 and P5 at all cochlear positions, including in the *Tecta* mutant background. Red radial lines and arcs represent the circular mean and circular standard deviation, respectively. N and n represent the number of animals and HCs analyzed, respectively. Bin size is 10°. A Watson U^2^ test is used to compare angular distributions across stages. All genotypes are littermate animals. Controls are *Pax2-Cre; Tecta^sec/+^; Vangl2^flox/+^*. *Tecta^sec/sec^* are *Pax2-Cre; Tecta^sec/sec^; Vangl2^flox/+^*. *Vangl2* cKO are *Pax2-Cre; Tecta^sec/+^; Vangl2^del/flox^*. *Tecta^sec/sec^; Vangl2* cKO are *Pax2-Cre; Tecta^sec/sec^; Vangl2^del/flox^*. Scale bars: 10 μm.

It remains elusive how the misoriented HC apical cytoskeleton, including the hair bundle, can be modified over time to eventually adopt a relatively normal lateral orientation. At postnatal stages, the distal tip of row 1 stereocilia in OHCs becomes embedded in the acellular tectorial membrane that covers the auditory epithelium. The tectorial membrane is instrumental in confining fluid flow and ensuring concerted deflection among neighboring hair bundles for proper mechano-electrical transduction (Goodyear and Richardson, 2018). The tectorial membrane was hypothesized to exert tractional forces through these stereocilia contacts to direct the normal refinement in HC orientation observed in the developing chick basilar papillae (Cotanche and Corwin, 1991). To investigate whether the tectorial membrane could impact the correction process, we crossed *Vangl2* cKO with an alpha-tectorin mutant (*Tecta^sec^*) in which the tectorial membrane fails to spread over the auditory epithelium and, thus, is not in contact with OHCs (Kim et al., 2019). No orientation defects were seen in *Tecta* mutants at P0, and the maturation of OHC3 orientation that normally occurs between P0 and P5 was not impacted (Fig. 2A,B). Severe OHC3 misorientation at P0 was largely comparable between single *Vangl2* and combined *Vangl2; Tecta* mutants, with the most striking disruptions, including inverted OHC3 pointing medially, at the cochlear mid and apex positions. By P5 however, both mutants showed a generally normal lateral OHC3 orientation at all cochlear positions (Fig. 2A,B). Based on these results, we conclude that HC reorientation is not affected by the loss of the overlying tectorial membrane. Moreover, since stereocilia imprints in the mouse tectorial membrane only appear around P5 (Verpy et al., 2011), the bulk of HC reorientation probably occurs before tectorial membrane attachment.

### Correction of hair cell orientation in absence of FZD3 and FZD6 proteins

**Fig. 3.**
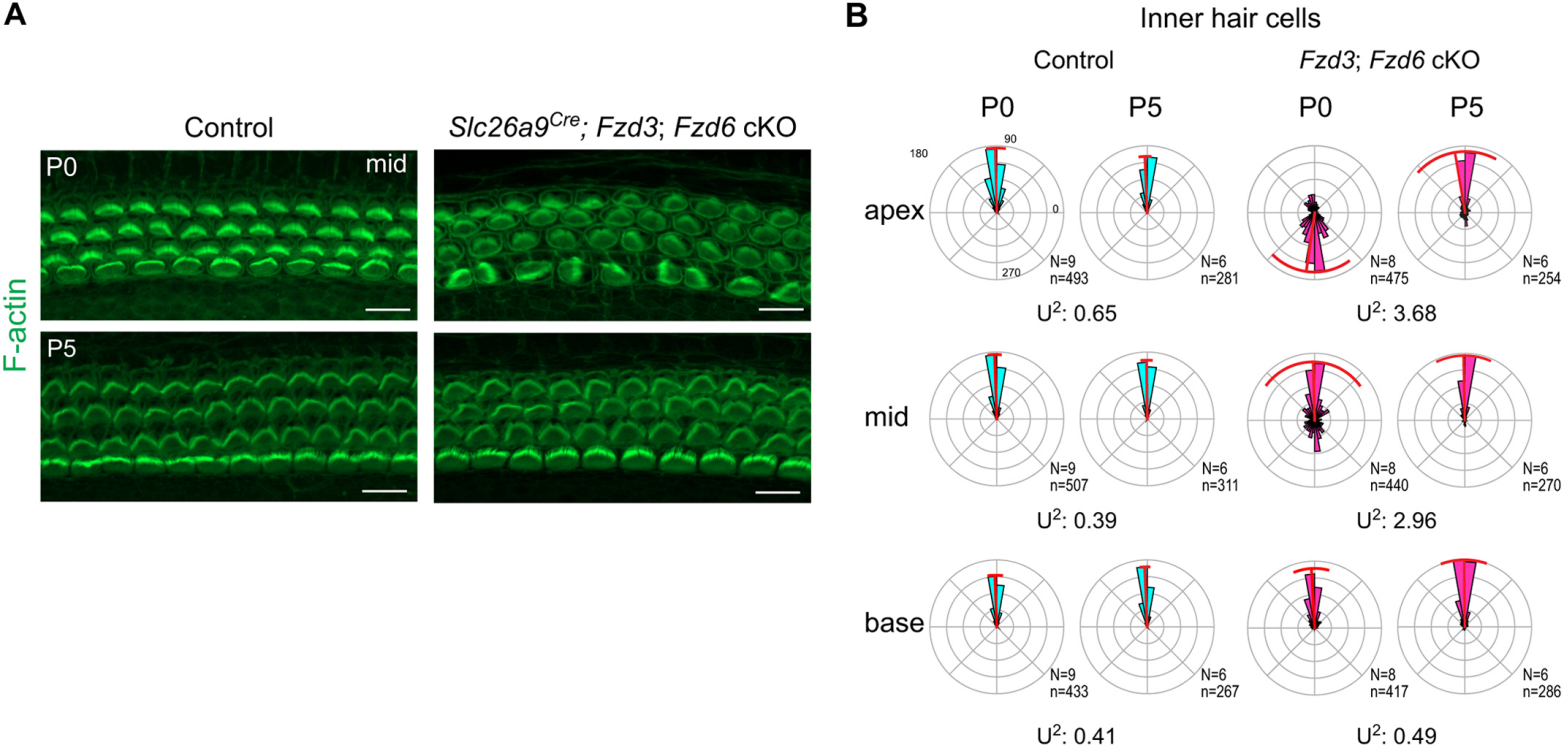
**Hair cell orientation in double *Fzd3; Fzd6* mutants at P0 and P5.** (A) Phalloidin (F-actin) labeling of the apical surface of the mouse auditory epithelium at P0 and P5 at the mid-cochlear position. (B) Circular histograms representing IHC orientation at the cochlear base, mid and apex positions. Note how IHC misorientation at the mid and apex positions at P0 is largely corrected at P5 in *Fzd3; Fzd6* cKO mutants. Red radial lines and arcs represent the circular mean and circular standard deviation, respectively. N and n represent the number of animals and HCs analyzed, respectively. Bin size is 10°. A Watson U^2^ test is used to compare angular distributions across stages. In B, data was pooled from *Emx2^Cre^*- and *Slc26a9^Cre^*-mediated inactivation of *Fzd3* (*Fzd3^del/flox^*) in a constitutive *Fzd6* mutant background. Control genotype is *Cre/+; Fzd3^flox/+^; Fzd6^del/+^.* Scale bars: 10 μm.

Severe HC orientation defects have also been reported in constitutive *Fzd3; Fzd6* double mutants at E18.5, most notably IHCs that are frequently inverted by 180° (Wang et al., 2006b). Similar to *Vangl2* mutants, these animals have severe neural tube closure defects and die at birth. To ask whether postnatal correction of misoriented HCs is specific to loss of VANGL1/2 or possibly a shared feature of core PCP mutants, we generated *Fzd3* and *Fzd6* double mutants that survive past birth. Constitutive *Fzd6* inactivation (Guo et al., 2004) and a constitutive *Fzd3* mutant allele (Wang et al., 2002) were combined with conditional *Fzd3* inactivation (Hua et al., 2013) using *Slc26a9^Cre^*. *Slc26a9* is expressed at the otic placode stage of inner ear development, and Cre-mediated recombination occurs throughout the inner ear and prior to HC specification (Urness et al., 2020) (*Slc26a9^Cre^; Fzd3^del/flox^; Fzd6^del/del^*, hereafter *Fzd3; Fzd6* cKO). We imaged cochlear IHCs at P0 and P5 at the cochlear base, mid and apex positions to obtain extended pseudo-time snapshots at each age since HC differentiation proceeds gradually from base to apex (Fig. 3A) (Lim and Anniko, 1985). At the P0 cochlear apex and mid positions, mutant IHCs were frequently inverted. This mirrors the *Fzd3; Fzd6* double knockout phenotype previously reported at E18.5 (Wang et al., 2006b). However, the more-mature IHCs at the base were oriented laterally at P0 in *Fzd3; Fzd6* cKO; by P5, IHCs along the entire length of the cochlea had orientations comparable to littermate controls (Fig. 3B). These results demonstrate that loss of FZD3 and FZD6 proteins, similar to loss of VANGL1 and VANGL2, results in temporary misorientation defects that are progressively and largely corrected during HC differentiation.

### Apical GPR156 enrichment in hair cells depends on the interacting partners VANGL1 and VANGL2

The core PCP machinery is unlikely to be involved in the correction process, since reorientation still occurs in *Vangl1; Vangl2* and *Fzd3; Fzd6* cKOs (Figs 1–3). Intriguingly, inactivating GPR156-GNAI activity, which is specific to HCs, results in a phenotype that resembles and is complementary to core PCP orientation defects. In *Gpr156* mutants, OHC1 and OHC2 have inverted orientations reminiscent of inverted OHC3 and IHC in *Vangl* and *Fzd* mutants (Jarysta et al., 2026; Kindt et al., 2021). This suggests that GPR156-GNAI signaling may be closely related to core PCP function in auditory HCs but, interestingly, HC misorientation is not corrected during postnatal development in *Gpr156* mutants (Kindt et al., 2021). To explore alternative mechanisms that could underlie the correction process in core PCP mutants, we examined whether the GPR156-GNAI reversal module might be involved.

**Fig. 4.**
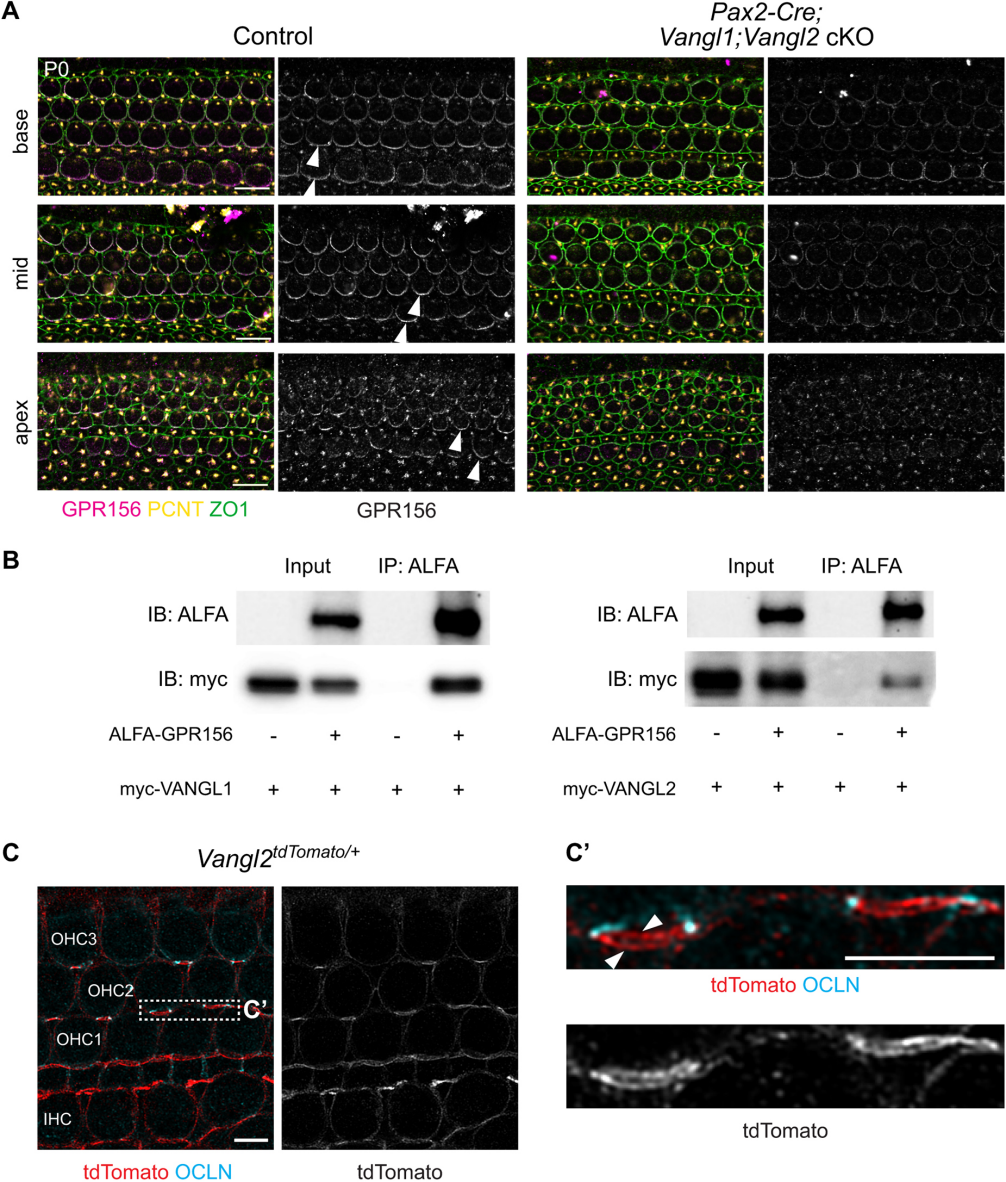
**Interaction between VANGL1 or VANGL2, and GPR156.** (A) GPR156 co-immunolabeling with pericentrin (PCNT) and ZO1 at the P0 cochlear base, mid and apex positions. Note how polarized enrichment of GPR156 at the medio-apical junction (arrowheads) is reduced in *Vangl1; Vangl2* cKO mutants. The GPR156 antibody non-specifically labels the pericentriolar region in some fields. Control is *Vangl1^del/flox^; Vangl2^del/flox^*. *Vangl1; Vangl2* cKO is *Pax2-Cre/+; Vangl1^del/flox^; Vangl2^del/flox^*. (B) Co-immunoprecipitation (IP) of myc-VANGL1 (left) and myc-VANGL2 (right) with ALFA-GPR156 in HEK293 cells. Results are representative of three experiments. (C) tdTomato and Occludin (OCLN) co-immunolabeling in a P0 *Vangl2^tdTomato^* heterozygote at the cochlear base. Area outlined in C is shown at higher magnification in C′. TCA fixation slightly detaches some cell-cell contacts and reveals tdTomato signals on both the OHC and supporting cell side (arrowheads), between four-way junctions labeled with OCLN. Scale bars: 10 μm in A; 5 μm in C and C′.

Evidence for an interaction between GPR156 and core PCP components was found in *Vangl2* (*Looptail*) mutants (*Vangl2^Lp^*), where the polarized enrichment of GPR156 at the apical HC junction was reduced in OHCs and retained yet abnormally positioned in IHCs (Kindt et al., 2021). These results were difficult to interpret and relate to normal protein function, because *Looptail* is a semi-dominant mutation (Kibar et al., 2001; Murdoch et al., 2001; Yin et al., 2012). We thus investigated how GPR156 responds to a clean loss of function in P0 *Vangl1; Vangl2* cKO mutants. At all cochlear positions, the medial crescent formed by GPR156 accumulation was either reduced or lost, irrespective of HC orientation revealed by the position of the pericentrin (PCNT)-labeled basal body that nucleates the kinocilium (Fig. 4A). This large dependence on VANGL proteins led us to consider whether GPR156 and VANGL1 or VANGL2 could physically interact in HCs. To test this idea, we used a heterologous assay and transfected HEK293 cells with ALFA-tagged GPR156 along with either myc-tagged VANGL1 or VANGL2, and immunoprecipitated GPR156 using a nanobody against the ALFA epitope. Both VANGL1 and VANGL2 co-precipitated with GPR156 (Fig. 4B), suggesting that GPR156 and VANGL proteins may also physically interact in cochlear HCs.

While GPR156 crescents occur in HCs, previous evidence suggested that VANGL2 was primarily enriched on the supporting cell side of the OHC-supporting cell junction (Giese et al., 2012; Montcouquiol et al., 2006). However, *Vangl2* is expressed in both cells types and these earlier results did not rule out the possibility that VANGL2 is present on both sides of that junction. To revisit this point, we used a *Vangl2* knock-in allele where tdTomato-VANGL2 faithfully recapitulates polarized protein distribution in different organ systems (Basta et al., 2021). Using a TCA fixation method that frequently loosens cell-cell contacts (Giese et al., 2012), we could detect the intracellular tdTomato reporter on both sides of OHC-supporting cell junctions (Fig. 4C). Together, these results suggest that GPR156, VANGL1 and VANGL2 may interact in HCs at the medial junction, and work together to regulate HC orientation. If so, GPR156-GNAI may participate in the postnatal reorientation process that rescues PCP phenotypes.

### GPR156 is reoriented ahead of the basal body during the correction process in *Fzd3; Fzd6* double mutants

**Fig. 5.**
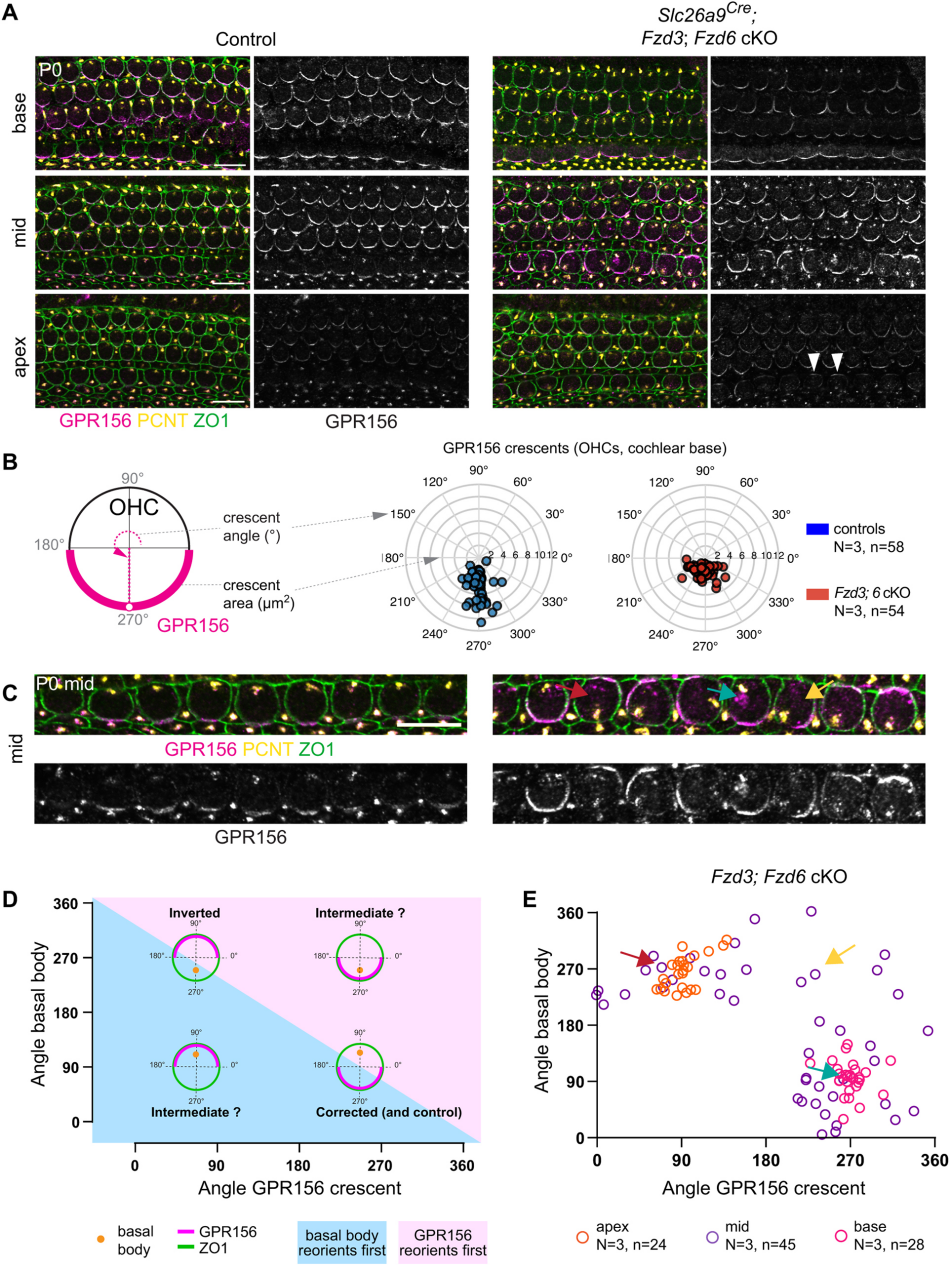
**GPR156 protein distribution in double *Fzd3; Fzd6* mutants.** (A) GPR156 co-immunolabeling with pericentrin (PCNT) and ZO1 at the P0 cochlear base, mid and apex positions. GPR156 is enriched at normal levels and the polarized GPR156 crescent is generally matching HC orientation, occurring opposite the basal body (PCNT). Arrowheads point to lateral GPR156 crescents in apical IHCs that are still misoriented in the mutant (medial basal body). Control is *Slc26a9^Cre/+^; Fzd3^flox/+^; Fzd6^del/+^* except for the base, which is *Slc26a9^Cre/+^; Fzd3^del/flox^; Fzd6^del/+^.* Mutant is *Slc26a9^Cre^; Fzd3^del/flox^; Fzd6^del/del^*. The GPR156 antibody non-specifically labels the pericentriolar region in some fields. (B) Quantification of GPR156 crescents at the cochlear base (A). Plots depict the orientation (0-360°; angular axis) and surface area (0-12 μm^2^; radial axis) of the crescents. In *Fzd3; Fzd6* cKOs, GPR156 crescents are shorter but remain medially oriented. (C) Higher magnification of the IHC region in A at the cochlear mid. (D) Diagram of IHC orientation states based on the angle adopted by the basal body (*y-*axis) and the GPR156 crescent (*x*-axis). Intermediate states between an inverted (top left) and normal/corrected (bottom right) IHC could theoretically include reorientation of the basal body ahead of GPR156 (bottom left) or reorientation of GPR156 ahead of the basal body (top right). (E) Actual plotting of IHCs lacking *Fzd3; Fzd6* at the cochlear base (magenta), mid (purple) and apex (orange) positions based on basal body and GPR156 crescent angles as in D. Note how transitioning IHCs at the cochlear mid (purple) map in the top right quadrant, suggesting that GPR156 reorients ahead of the basal body. In C and E, arrows indicate inverted (red), intermediate (yellow) or corrected (green) IHCs. N and n represent the number of animals and HCs analyzed, respectively. Scale bars: 10 μm.

We next considered how loss of FZD3 and FZD6 affects GPR156 distribution and found that, unlike in *Vangl1; Vangl2* cKOs, GPR156 was initially enriched in normal amounts at the HC apical junction in the P0 *Fzd3; Fzd6* cKO cochlea (Fig. 5A). As reported previously (Jia et al., 2023; Kindt et al., 2021), the polarized distribution of GPR156 generally matched HC orientation, being opposite from the basal body and/or kinocilium. Before correction (P0 apex), GPR156 formed a crescent along the lateral edge of medially oriented IHCs in *Fzd3; Fzd6* cKOs (Fig. 5A, arrowheads), contrasting with a medial crescent in laterally oriented control IHCs. Following correction in the *Fzd3; Fzd6* cKO (P0 base), GPR156 was found along the medial edge of laterally oriented IHCs, as expected (Fig. 5A). However, GPR156 formed partial crescents that remained constrained to the medial HC half but showed variable distributions compared to controls (Fig. 5A,B). We conclude that maintaining the full span of GPR156 at the medial junction does require FZD3 and FZD6, unlike early establishment (P0 apex).

To approximate the behavior of GPR156 during the correction process, when the basal body is relocating from a medial to a lateral position, we focused on IHCs at the P0 cochlear mid region (Fig. 5A,C). There, a wide range of IHC orientations were observed in *Fzd3; Fzd6* cKO and likely correspond to cells caught at various stages of reorientation. Interestingly, at this cochlear position, we observed inverted IHCs where the basal body and the GPR156 crescent were both located on the medial side of the IHC (Fig. 5C, yellow arrow), suggesting that GPR156 and the apical IHC cytoskeleton do not always correct their orientation in concert. To determine whether the position of the GPR156 crescent is typically corrected before or after the basal body is repositioned, we measured and graphed the orientation of both labels at the mid-cochlea.

Normal control IHCs or corrected IHCs in *Fzd3; Fzd6* cKOs (P0 base) have a laterally positioned basal body (∼90°) and a medially positioned GPR156 crescent (∼270°; Fig. 5D,E; magenta points in E). Those positions are reversed before correction at the *Fzd3; Fzd6* cKO P0 apex (basal body, ∼270°; GPR156, ∼90°; orange points in E). If the basal body relocalized first, we predicted that ‘intermediate’ IHCs fixed during the correction process would include examples where both the basal body and GPR156 are in lateral positions (∼90°; Fig. 5D). Alternatively, if the GPR156 crescent relocalized first, then intermediate IHCs would include examples where both the basal body and GPR156 are medial (∼270°). When plotting the angular position of the basal body against the position of GPR156 for IHCs from the apex, mid and basal positions in *Fzd3; Fzd6* cKOs, we observed that reorienting IHCs at the cochlear mid (Fig. 5E, purple points) were found only in the top-right quadrant of the graph. These points corresponded to cells in which the angular position of the basal body and GPR156 crescent were ∼270° and both were observed on the medial side of the IHC. In contrast, there were no intermediate cells plotted in the opposite quadrant, indicating that the reorientation of GPR156 crescents typically occurs ahead of the basal body during correction.

### Correction of HC orientation occurs *ex vivo* in cochlear culture

**Fig. 6.**
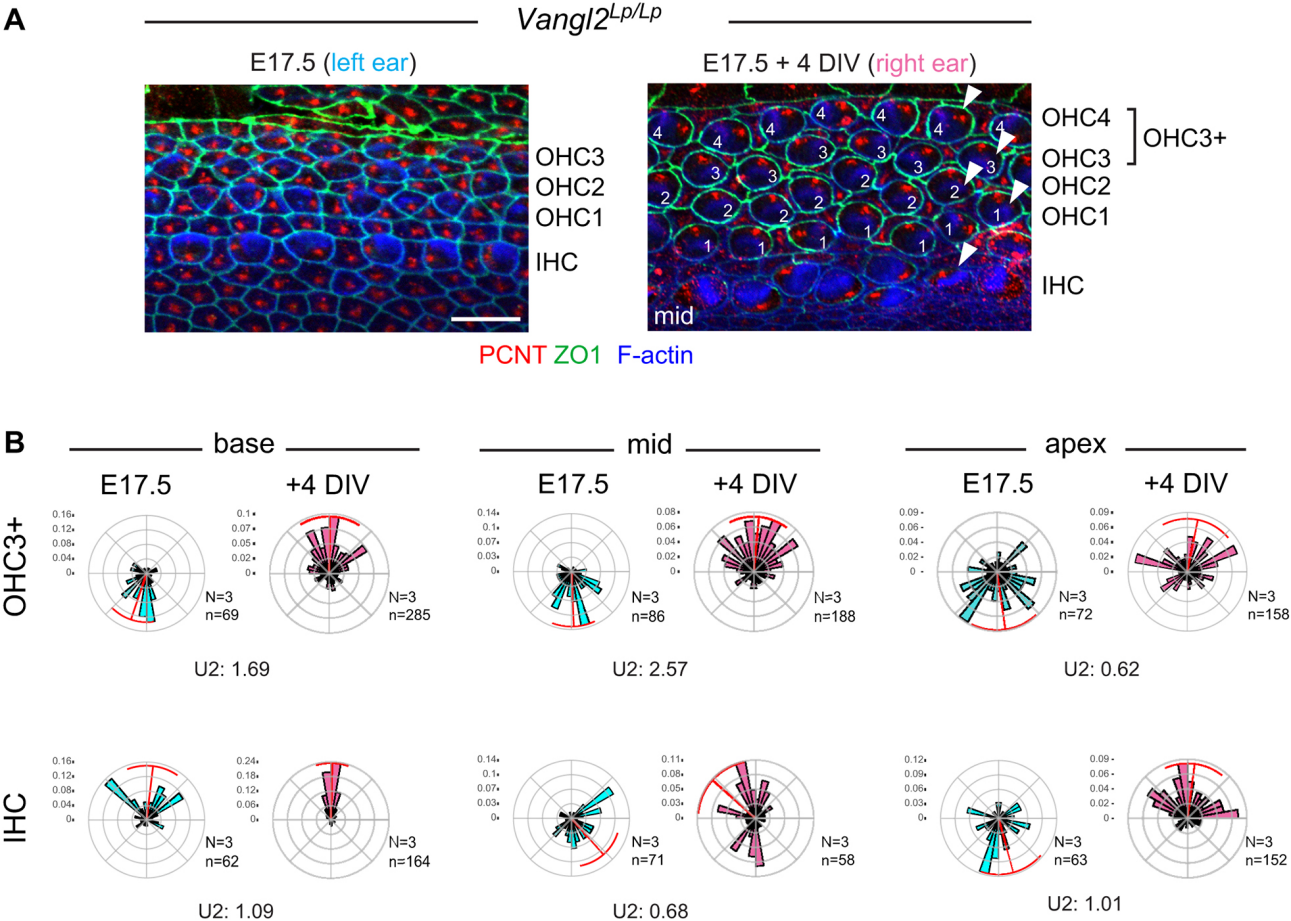
**Hair cell reorientation *ex vivo* in *Vangl2^Lp^* cochlear explants.** (A) Illustrative example of a *Vangl2^Lp^*^/*Lp*^ left inner ear fixed at E17.5 (left) and a right inner ear from the same animal cultured for 4 days *in vitro* (DIV) (right). Mid-cochlear samples were immunolabeled for pericentrin (PCNT, basal body), ZO1 and phalloidin (F-actin) to derive HC orientation. HC row organization is disrupted in cultured *Vangl2^Lp^* mutants and OHC row attribution is indicated. OHC3 and OHCs in further extra rows are collectively indicated as OHC3+. (B) Circular histograms representing IHC and OHC3+ orientation at the cochlear base, mid and apex positions in *Vangl2^Lp/Lp^* mutants. Note how OHC3+ at all positions and IHCs at the mid and apex are frequently misoriented medially at E17.5, but reorient laterally after culture. Red radial lines and arcs represent the circular mean and circular standard deviation, respectively. N and n represent the number of animals and HCs analyzed, respectively. Bin size: 10°. A Watson U^2^ test is used to compare angular distributions across stages. Scale bar: 10 μm.

Next, we investigated whether GPR156-GNAI signaling may be involved in correcting HC orientation in core PCP mutants. Our goal was to inactivate GPR156-GNAI signaling in core PCP mutants and examine whether HC reorientation would be affected. As it was unrealistic to breed *Gpr156^del^* with either the *Vangl1; Vangl2* cKO or *Fzd3; Fzd6* cKO models, we used pertussis toxin to block GNAI signaling in core PCP mutants (Kindt et al., 2021). Since the correction of orientation can occur in the absence of an overlying tectorial membrane (Fig. 2), we reasoned that the correction may also be studied in 2D cochlear cultures. To test whether reorientation and phenotype rescue occurred *ex vivo*, we first used the semi-dominant *Vangl2^Lp^* stain in which homozygotes show severe HC misorientation (Montcouquiol et al., 2003). *Vangl2^Lp/Lp^* mutants and littermate controls were harvested at E17.5, and the left cochlea immediately fixed to assess misorientation at this stage. The right cochlea was explanted and cultured for 4 days as an open-book preparation to examine reorientation potential *ex vivo*. Consistent with previous reports, E17.5 *Vangl2^Lp^*^/Lp^ mutants showed HC misorientation that was most severe in OHC3 (all cochlear positions) and IHCs (cochlear mid and apex) (Fig. 6A,B) with HCs in these rows trending towards a full 180° inversion. However, after 4 days in culture (a stage equivalent to ∼P2 *in vivo*), IHC and OHC3 orientation was strikingly improved, switching to a more lateral orientation in all OHC3 and in IHCs at the mid and apex positions (Fig. 6B). Thus, HCs can correct their orientation at perinatal stages in the semi-dominant *Vangl2^Lp^* mutant similar to the recessive *Vangl1; Vangl2* cKO (Figs 1 and 2), and the reorientation process can be recapitulated in organotypic culture conditions.

### Blocking GPR156-GNAI signaling facilitates correction of HC orientation in core PCP mutants

**Fig. 7.**
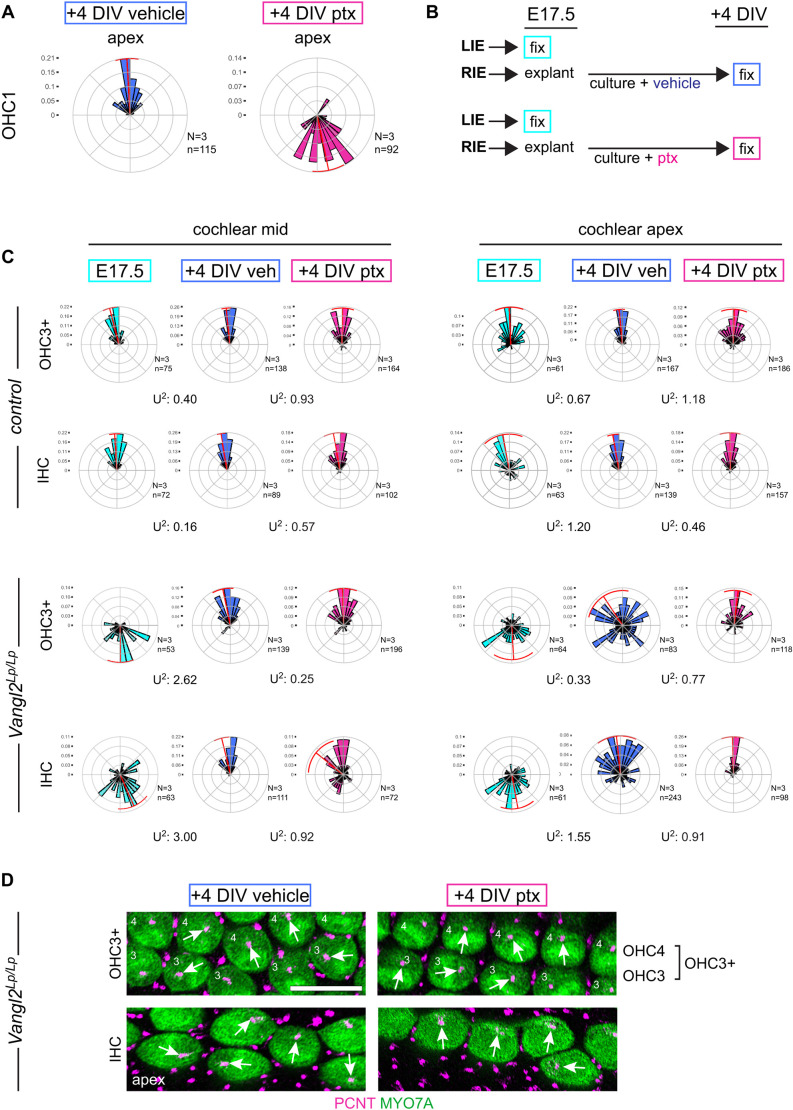
**Hair cell reorientation *ex vivo* in *Vangl2^Lp^* cochlear explants upon blocking GPR156-GNAI signaling.** (A) Circular histograms representing OHC1 orientation at the cochlear apex after culture to validate pertussis toxin (ptx) activity (control samples). Ptx, but not vehicle exposure, inverts OHC1, as reported previously. (B) Experimental design, which also applies to *Fzd3; Fzd6* cKO experiments in Fig. 8. E17.5 control and *Vangl2^Lp/Lp^* fetuses are processed similarly, with the left inner ear (LIE) fixed and the right inner ear (RIE) cultured for 4 days *in vitro* (DIV). During culture, explants are exposed to either vehicle (glycerol) or ptx (10 ng/ml). Cyan indicates E17.5 samples (no culture); blue indicates E17.5+4 DIV samples exposed to vehicle; magenta indicates E17.5+4 DIV samples exposed to ptx. (C) Circular histograms representing IHC and OHC3+ orientation at the cochlear mid and apex positions. Control HCs (top) show a generally normal lateral orientation regardless of stage and condition. Blocking GPR156-GNAI (ptx) inverts OHC1-2 (A) but does not severely affect IHC or OHC3 orientation (C), as shown previously. *Vangl2^Lp^* mutant IHCs and OHC3 are severely misoriented at E17.5, but a lateral reorientation is observed after culture with vehicle (blue). ptx seems to help promote lateral reorientation at the cochlear apex (magenta). (D) Illustrative examples of OHC3 and further rows (OHC3+), and IHCs after culture at the cochlear apex. Explants were immunolabeled for pericentrin (PCNT, basal body) and MYO7A (HC) to derive HC orientation (arrows). HC row organization is disrupted in cultured *Vangl2^Lp^* mutants and OHC row attribution is indicated. Red radial lines and arcs in A and C represent the circular mean and circular standard deviation, respectively. N and n represent the number of animals and HCs analyzed, respectively. Bin size: 10°. A Watson U^2^ test is used to compare angular distributions between E17.5 and +4 DIV (vehicle), and between +4 DIV (vehicle) and +4 DIV (ptx). Controls are pooled *Vangl2^+/+^* and *Vangl2^Lp/+^* samples. Scale bar: 10 μm.

GPR156 signals through GNAI to influence HC orientation and, consequently, expression of pertussis toxin (ptx) catalytic subunit (ptxA) in HCs closely recapitulates the misorientation profile observed upon *Gpr156* inactivation in the cochlea and macular organs (Kindt et al., 2021; Tarchini et al., 2013, 2016). We therefore blocked GPR156-GNAI signaling by adding ptx holoenzyme (10 ng/ml) to the medium during cochlear culture of *Vangl2^Lp^* and control explants. To confirm ptx activity, we measured OHC1 orientation after 4 days in control cochlear cultures because this HC row is most impacted in the *Gpr156* mutant. As expected from previous results using this culture approach (Ezan et al., 2013) and from ptxA usage *in vivo* (Kindt et al., 2021; Tarchini et al., 2013, 2016), the vast majority of OHC1 in the cochlear apex were inverted by ptx treatment, whereas vehicle exposure resulted in normal lateral OHC1 orientation (Fig. 7A). Next, we cultured the right cochlea of E17.5 *Vangl2^Lp/Lp^* and littermate controls in the presence of ptx, and compared IHC and OHC3 orientation at the cochlear mid and apex positions after 4 days to the vehicle control condition (Fig. 7B). As before, left cochleae were fixed at E17.5 to reveal baseline misorientation (Fig. 7B, cyan). As expected from previous studies, ptx had little impact on IHC or OHC3 orientation in control cochleae after 4 days *in vitro* (Fig. 7C, magenta). The severe OHC3 and IHC misorientation in E17.5 *Vangl2^Lp/Lp^* was partially corrected during the culture period (Fig. 7C,D, blue), as observed previously (Fig. 6). Comparing *Vangl2^Lp/Lp^* tissues cultured in vehicle- and ptx-treated conditions, we noted a greater improvement in orientation for IHCs and OHC3 at the apex for explants treated with ptx. These HCs more consistently adopted a lateral (90°) orientation reminiscent of similarly positioned HCs cultured under control conditions (Fig. 7C,D).

**Fig. 8.**
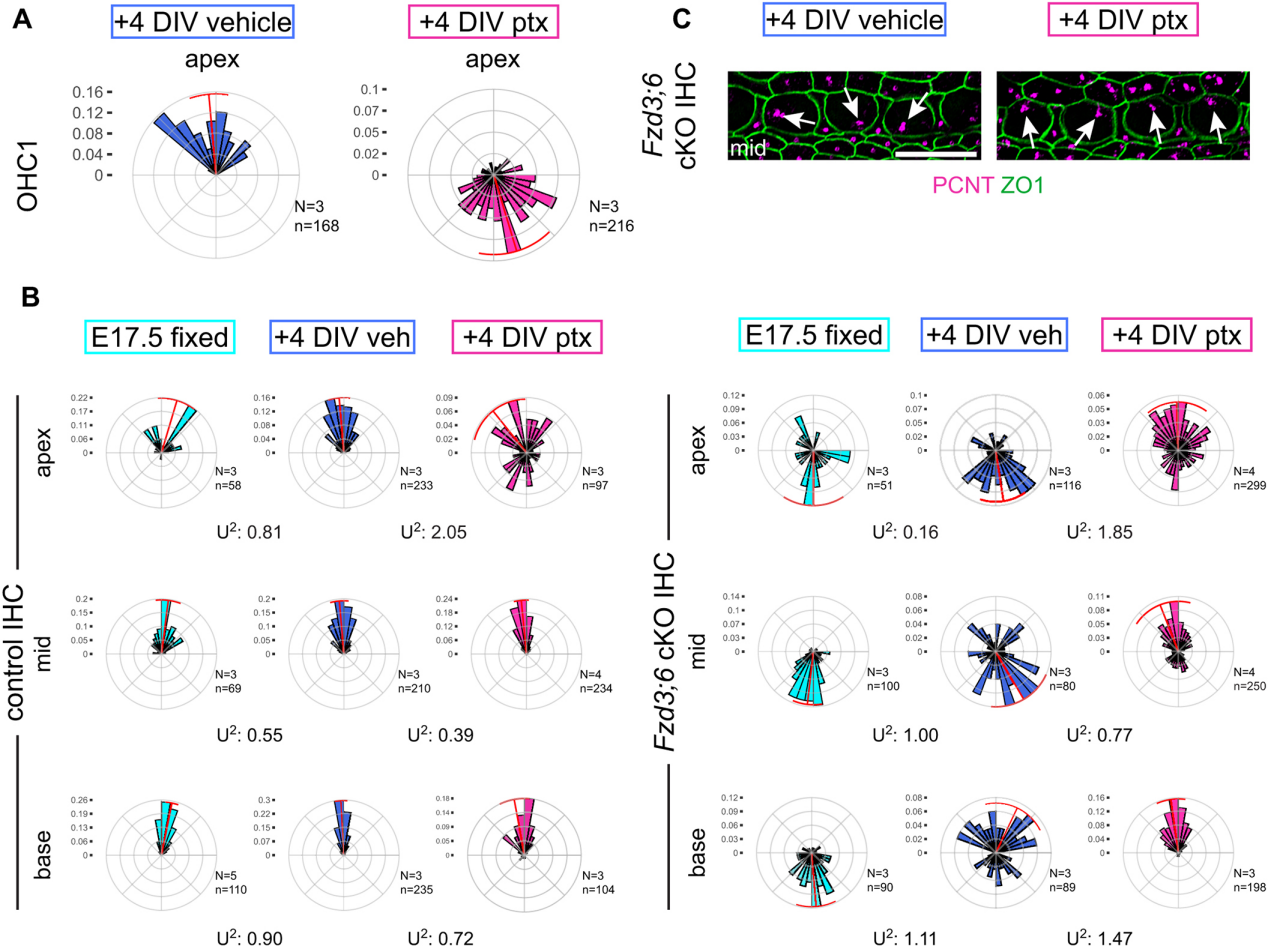
**Inner hair cell reorientation *ex vivo* in *Fzd3; Fzd6* cKO cochlear explants upon blocking GPR156-GNAI signaling.** (A) Circular histograms representing OHC1 orientation at the cochlear apex after culture to validate ptx activity (cKO samples). Note how ptx, but not vehicle, exposure largely inverts OHC1, as reported previously. (B) Circular histograms representing IHC orientation at the cochlear base, mid and apex positions. Control IHCs (left) show a largely normal lateral orientation except upon ptx exposure at the cochlear apex. *Fzd3; Fzd6* cKO mutant IHCs (right) are severely misoriented at E17.5, and a modest degree of lateral reorientation is observed after culture with vehicle (blue), particularly at the cochlear base. ptx strongly enhances lateral reorientation at all cochlear positions (magenta). Controls are *Slc26a9^Cre/+^; Fzd3^flox/+^; Fzd6^del/+^*. *Fzd3; 6* cKO are *Slc26a9^Cre/+^; Fzd3^del/flox^; Fzd6^del/del^*. (C) Illustrative examples of IHCs after culture at the cochlear mid. Explants were immunolabeled for pericentrin (PCNT, basal body) and ZO1 to derive HC orientation (arrows). In A and B, red radial lines and arcs represent the circular mean and circular standard deviation, respectively. N and n represent the number of animals and HCs analyzed, respectively. Bin size: 10°. A Watson U^2^ test is used to compare angular distributions between E17.5 and +4 DIV (vehicle), and between +4 DIV (vehicle) and +4 DIV (ptx). Scale bar: 10 μm.

To assess the effect of blocking GPR156-GNAI signaling in the *Fzd3; Fzd6* cKO where GPR156 enrichment is retained (Fig. 5), we used the same experimental approach (Fig. 7B) with *Fzd3; Fzd6* cKO explants harvested at E17.5. We confirmed ptx activity by monitoring OHC1 orientation as before (Fig. 8A), and focused analyses on IHCs that are most severely affected in *Fzd3; Fzd6* cKO (Fig. 3A,B) (Wang et al., 2006b). In normal culture conditions (vehicle), we observed some degree of correction for IHCs at the cochlear base and mid positions after 4 days in culture (Fig. 8B), but correction was less complete than observed *in vivo* between P0 and P5 (Fig. 3B). The extent of correction was also less than for IHCs in cultured *Vangl2^Lp^* mutants (Figs 6B and 7C,D). Cultured IHCs at the cochlear apex did not appear to reorient in *Fzd3; Fzd6* cKO explants (Fig. 8B) as they do *in vivo* (Fig. 3). Remarkably, however, ptx exposure led to an improvement in the reorientation process, with a greater number of IHCs adopting lateral orientations compared to vehicle control conditions (Fig. 8B,C). Furthermore, apical IHCs that did not reorient in control culture conditions did so to some extent upon ptx exposure (Fig. 8B). This result is consistent with improved correction observed in *Vangl2^Lp^* mutant HCs following ptx-treatment (Fig. 7C).

In summary, the redistribution of the GPR156 crescent ahead of the basal body in *Fzd3; Fzd6* cKO (Fig. 5C-E) is not likely part of an instructive role for GPR156-GNAI signaling in the reorientation process. Instead, maintained GPR156-GNAI activity in *Fzd3; Fzd6* cKOs (Fig. 5A) and residual activity in *Vangl* mutants (Fig. 4A) both appear to counteract or delay the reorientation process. Based upon these *ex vivo* experiments and considering GPR156-VANGL protein interactions (Fig. 4A,B), we conclude that GPR156 works in parallel to core PCP signaling to define proper HC orientation.

## DISCUSSION

Previous work in conditional *Vangl2* mutants showed that misoriented auditory HCs can correct their orientation over time (Copley et al., 2013). That study also revealed that the distribution of VANGL1, FZD6 and prickle-like 2 (PK2) is disrupted during the correction process, suggesting that partner core PCP proteins are unlikely to drive reorientation. Here, we confirm this hypothesis by showing that reorientation still occurs in *Vangl1; Vangl2* and *Fzd3; Fzd6* double mutants. These findings indicate that impaired VANGL and FZD function leads only to transient HC misorientation. This suggests that an alternative pathway is responsible for reorienting HCs at later developmental stages, at least in this mutant context. This is reminiscent of hair follicles in *Fzd6* and *Vangl2* mutants, which initially appear misoriented but undergo postnatal correction (Cetera et al., 2017; Wang et al., 2006a, 2010). Both systems seem to involve temporally and mechanistically distinct alignment processes but direct comparisons are challenging since one governs a multicellular structure and the other effects individual HCs.

Early HC orientation results from the off-center migration of the basal body and kinocilium as HCs first break planar symmetry. The direction of this migration is thought to be controlled by opposing core PCP complexes (Montcouquiol et al., 2003). We propose that this early core PCP-based process defines auditory HC orientation up to around birth (E18.5-P0), after which a distinct mechanism might become involved at postnatal stages. We envision two broad scenarios: (1) the later mechanism may be active during normal development, and responsible for refining HC orientation after birth. One study indeed reported more tightly lateral HC orientation at P10 compared to P0 in the mouse cochlea (Dabdoub et al., 2003). In this scenario, the later mechanism would operate across a broad range, capable of directing both subtle reorientation (<∼5°) during normal development and extensive reorientation (<180°) in severe core PCP mutants. Alternatively, (2) the late mechanism may be specifically activated in mutant contexts and distinct from a potential refinement mechanism. Notably, reoriented HCs are not as precisely aligned as controls (Figs 1, 2 and 4) and adult HCs at the cochlear apex remain misoriented in the absence of VANGL2 (Copley et al., 2013). This suggests that the late mechanism operates within a broad but finite developmental window and cannot, on its own, achieve the precise adult hair bundle organization expected from normal development. A natural refinement process would therefore require the early core PCP protein function. In either scenario, the pathway responsible for reorientation must act in parallel to core PCP. In *Drosophila*, the Fat-Dachsous protocadherin pathway plays such a parallel role, but there is limited evidence for Fat proteins influencing mouse HC orientation (Saburi et al., 2008, 2012). Another potential candidate is non-canonical Wnt signaling, which has been proposed to act in parallel to core PCP in the auditory organ but primarily influences cochlear duct elongation over HC orientation (Landin Malt et al., 2020; Najarro et al., 2020; Xie et al., 2024). Notably, cochlear length was unaffected when *Vangl2* inactivation was limited to the inner ear (Copley et al., 2013); similarly, no obvious elongation defects were observed in *Vangl1; Vangl2* or *Fzd3; Fzd6* mutants.

Importantly, postnatal reorientation in core PCP mutants does not depend on the GPR156-GNAI reversal module. Instead, GPR156-GNAI appears to signal alongside core PCP proteins at earlier stages of polarization because (1) GPR156 binds to and depends on VANGL1/2 for its normal polarized enrichment (Fig. 4), (2) GPR156-GNAI opposes corrective reorientation of OHC3 and IHC in cochlear explants (Figs 7–8), and (3) *Gpr156* inactivation results in severely misoriented HCs with normally patterned apical cytoskeletons, similar to core PCP mutants (Kindt et al., 2021). In summary, the molecular identity of the later orientation mechanism uncovered in core PCP mutants remains unknown. In multiciliated cells, directional fluid flow acts in parallel with core PCP to organize the distribution of microtubule-based cilia and their aligned beating (Guirao et al., 2010). By analogy, the correction of HC orientation in core PCP mutants may similarly rely on mechanical cues. These forces would need to be intrinsic to the cochlear floor epithelium, as correction occurs both *in vivo* without tectorial membrane coverage (Fig. 2) and *ex vivo* in cochlear cultures (Figs 6–8).

It is remarkable that *Vangl* and *Fzd* mutants share with *Gpr156* mutants a misorientation phenotype that initially appears as a ∼180° inversion. This observation would suggest that HCs appearing randomly oriented in core PCP mutant cochleae are actually cells that were undergoing lateral reorientation at tissue harvesting time. In contrast, core PCP disruption in other epithelial systems, including vestibular organs, often results in randomized HC orientation without a correction process (Copley et al., 2013). Given the comparable role for GPR156-GNAI in orienting HCs, it remains unclear why OHC1-2 fail to reorient over time in *Gpr156* mutants (Kindt et al., 2021). This discrepancy might reflect the anatomical location of OHC1-2 within the auditory epithelium, rather than a fundamental difference between core PCP and GPR156 function. For example, unlike IHCs and OHC3, OHC1-2 may be physically constrained from substantially reorienting (from medial to lateral, ∼180°) due to unique adhesion properties between OHC1-2 and supporting cell neighbors (outer pillar and Deiter's 1-2 cells). Nevertheless, OHC1-2 can still improve the accuracy of their lateral orientation in *Vangl1; Vangl2* cKOs (Fig. 1B). Alternatively, it is possible that HCs in PCP mutants are more amenable to reorientation than in *Gpr156* mutants. For example, VANGL1 or VANGL2 have been proposed to both positively and negatively regulate intercellular junctions or adhesion (Lindqvist et al., 2010; Nagaoka et al., 2014).

It also remains unclear why different proteins selectively affect specific HC subtypes when defective. Loss of VANGL1 and VANGL2 or FZD3 and FZD6 show defects most severe in OHC3 and IHCs. In contrast, inactivating GPR156-GNAI signaling principally affects OHC1-2. This selective vulnerability is unexpected given that GPR156 protein is similarly polarized at the medio-apical junction across IHCs and all three OHC rows. By comparison, asymmetric distribution of core PCP proteins, including VANGL1 and VANGL2, and FZD3 and FZD6, occurs in both HCs and supporting cells, although their precise localization pattern remains incompletely resolved (Kindt and Tarchini, 2025; Tarchini and Lu, 2019). The intricate cellular architecture of the auditory epithelium in which four distinct HC types are surrounded by different combinations of seven types of supporting cells may contribute to these subtype-specific phenotypes. The diversity of junctions formed between these cell types could influence how polarity cues are interpreted or propagated, thereby shaping the differential impact of core PCP pathway disruptions.

VANGL and FZD are typically segregated to opposite sides of the apical junction within a single cell, with CELSR present in both complexes and bridging them across neighboring cells (Aw and Devenport, 2017). This arrangement was generally thought to be conserved in the mouse auditory epithelium. Mosaic analysis demonstrated that FZD6 is at the medial side of the HC (Wang et al., 2006b), and VANGL2 has been proposed to be primarily on the lateral side of the adjacent supporting cell (Giese et al., 2012; Montcouquiol et al., 2006), with CELSR1 possibly bridging the two (Duncan et al., 2017). Here, we find that tdTomato-VANGL2 is enriched on both sides and that VANGL1 and VANGL2 bind GPR156 in a heterologous system. As *Gpr156* is exclusively expressed in HCs, we propose that VANGL2 and GPR156 interact at the medial junction in HCs. One hypothesis is that VANGL, FZD and GPR156, when localized specifically at the medial HC junction, all function in the normal reversal process that establishes lateral auditory HC orientation (Tarchini, 2021). This could explain why phenotypes in *Vangl*, *Fzd* and *Gpr156* mutants trend towards inversion rather than randomization. In this light, the corrective reorientation observed in *Vangl* and *Fzd* mutants may represent a delayed reversal. This may also explain why auditory HCs are more amenable to correction than other cell types affected by core PCP disruption.

It is not necessarily surprising to observe VANGL2 enrichment on both sides of the HC-supporting cell junction. This is consistent with a recent study using STED super-resolution microscopy that showed that trans-cellular core PCP complexes can adopt either orientation across the same junction (FZD>VANGL or VANGL>FZD), with overall polarization arising from a bias in their proportion (Basta et al., 2025). Interestingly, the lateral HC junction does not show enrichment of either VANGL1 and VANGL2, or FZD3 and FZD6, deviating from canonical core PCP patterning. This further suggests that VANGL and FZD signaling in the auditory epithelium may involve unique molecular activities distinct from those in other epithelial systems.

## MATERIALS AND METHODS

### Mouse strains and husbandry

The following individual mouse strains were used in these studies: *Emx2^Cre^* (MGI:3579416) (Kimura et al., 2005), *Fzd3^del^* (MGI: 2388632) (Wang et al., 2002), *Fzd3^flox^* (MGI: 5510964) (Hua et al., 2013), *Fzd6^del^* (MGI: 3050103) (Guo et al., 2004), *Pax2-Cre* (MGI:3046196) (Ohyama and Groves, 2004), *Slc26a9^Cre^* (MGI:6715244) (Urness et al., 2020), *Tecta^Sec^* line (Kim et al., 2019), *Vangl1^flox^* (MGI: 5440498) (Chang et al., 2016), *Vangl2^del^* (MGI: 5430219) (Murdoch et al., 2001; Yin et al., 2012), *Vangl2^flox^* (MGI: 5430220) (Copley et al., 2013), *Vangl2^LPT/LeJ^* (MGI: 1857642) and *Vangl2^tdTomato^* (MGI: 7382795) (Basta et al., 2021).

*Vangl1; Vangl2* conditional knockouts were produced by crossing *Pax2-Cre/+*; *Vangl1^del/+^; Vangl2^del/+^* males with *Vangl1^flox/flox^; Vangl2^flox/flox^* females, as described previously (Stoller et al., 2018). *Vangl2*; *Tecta^sec^* double mutants were produced by crossing *Pax2-Cre/+; Tecta^sec/+^*; *Vangl2^del/+^* males with *TectA^sec/sec^; Vangl2^flox/flox^* females. The *Fzd3; Fzd6* conditional knockouts were produced by crossing *Emx2^Cre/+^* or *Slc26a9^Cre/Cre^*; *Fzd3^del/+^*; *Fzd6^del/+^* males with *Fzd3^flox/flox^*; *Fzd6^del/del^* females, as described previously (Ghimire and Deans, 2019). *Emx2*^Cre^ mediated recombination initiates at the otic vesicle stage of development (Goodrich and Deans, 2024) and spans the entire developing cochlea. Since both *Emx2^Cre^* and *Slc26a9^Cre^* are expressed prior to HC specification, some *Fzd3; Fzd6* cKOs generated using either Cre were pooled for analysis, as indicated. The semi-dominant *Vangl2^LPT/LeJ^* line was obtained at The Jackson Laboratory and bred to C57BL6/J to mitigate background-dependent imperforate vagina issues rendering heterozygote females infertile. Mouse samples analyzed ranged from E17.7 to P5, as indicated in each figure, and included both males and females. As single *Vangl1*, *Fzd3* and *Fzd6* mutants do not show obvious phenotypes in the auditory epithelium, they were sometimes used as controls, where indicated. Animals were maintained under standard housing conditions and all animal work was reviewed for compliance and approved by the Animal Care and Use Committee of The Jackson Laboratory and University of Utah.

### Immunolabeling

Temporal bones were extracted and either fixed in 4% PFA prepared in 67 mM Sorensons' phosphate buffer for 2 h on ice (Figs 1–3), fixed in 10% trichloroacetic acid (TCA) (Sigma T8657) in water for 10 min on ice (Figs 4, 5, 7 and 8), or fixed in 4% PFA in PBS for 1 h at 4°C (Fig. 6) before being micro-dissected to expose the sensory epithelium. Samples were then permeabilized and blocked in PBS with 0.5% Triton-X100 and bovine serum albumin (1%) for at least 1 h at room temperature. Primary and secondary antibodies were incubated overnight at 4°C in PBS and, when used, dye-conjugated phalloidin was added with secondary antibodies. After each antibody incubation, samples were washed three times with PBS+0.05% Triton-X100 before a final post-fixation in PFA 4% for 1 h at room temperature. Samples were then mounted flat on a charged slide and mounted under a 18×18 mm #1.5 coverglass (VWR 48366-045). Prolong Gold AntiFade Mountant (Invitrogen P36934) or Mowiol (Calbiochem/MilliporeSigma 4759041) were used as mounting medium. Mowiol (10% w/v) was prepared in 25% (w/v) glycerol and 0.1 M Tris-Cl (pH 8.5).

Primary antibodies used were: goat anti-GPR156 (SCBT, sc-102572; TCA fixation; 1:200); rabbit anti-pericentrin (PCNT) (Biolegend/Covance, PRB-432C; PFA or TCA; *Vangl1; Vangl2* cKO in Fig. 4, *Fzd3; 6* ckO in Fig. 5 and *Vangl2^Lp^*experiments in Figs 6 and 7; 1:400); rabbit anti-pericentrin (PCNT) (Abcam, ab4448; TCA; *Fzd3; Fzd6* cKO experiments in Fig. 8; 1:1000); rat anti-ZO1 (Developmental Studies Hybridoma Bank, R26.4C; TCA or PFA; 1:20); mouse anti-MYO7 (Developmental Studies Hybridoma Bank, MYO7A 138-1; TCA; 1:400); mouse anti-β2-spectrin (SPTBN2) (BD Biosciences, 612563; TCA; 1:200); rabbit anti-RFP (Rockland 600-401-379; TCA; 1:200) to detect tdTomato; mouse anti-myc (GenScript, A00704; co-IP in Fig. 4; 1:1000); and recombinant anti-ALFA Nb Nluc (Porter et al., 2025; co-IP in Fig. 4; 2 µg/ml).

Secondary antibodies from ThermoFisher Scientific were raised in donkey and conjugated to Alexa Fluor (AF) 488, 555 or 647 [donkey anti-rat AF488 (A-21208; 1:1000), donkey anti-goat AF555 (A-21432; 1:1000), donkey anti-rabbit AF555 (A-31572; 1:1000), AF647 (A-31573; 1:500), donkey anti-mouse AF647 (A-31571; 1:500)]. Donkey anti-rabbit Cy3 (Jackson ImmunoResearch 711-165-152; 1:1000) was used for *Fzd3;6* explants in Fig. 8. Fluorescent conjugated phalloidins used to reveal F-actin were from ThermoFisher Scientific (AF488; A12379) (Figs 1–3) and Biotium (CF405, 89138-126) (Fig. 6).

### Cochlear cultures

Inner ears from *Vangl2^Lp^* or *Fzd3; Fzd6* cKO animals and control littermates were harvested at E17.5 in HBSS (Gibco, 14065-056) with 5 mM Hepes (Gibco, 15630-080). The left inner ear was immediately fixed either in 4% PFA (Fig. 6) or in TCA for 10 min on ice (Figs 7 and 8), then stored in PBS at 4°C until further dissection. After removing the condensed mesenchyme off the right cochlea, the intact cochlear duct was separated from the modiolus and cut into three segments at ∼33% and ∼66% from the base to produce three explants: base, mid and apex. For each explant, Reissner's membrane was opened and the lateral wall, still attached to the cochlea, was flipped open to expose the auditory epithelium. These ‘open book’ preparations were explanted on tissue culture grade 35 mm dishes (CellTreat, 1228K66) coated with poly-D-lysine (10 μg/ml; MP Biomedicals, ICN10269410). The explants were then cultured for 4 days in DMEM/F12 (Corning, 10-090-CV) with 10% fetal bovine serum and ciprofloxacin (10 μg/ml; Sigma-Aldrich, 17850). Pertussis toxin (ptx; 10 ng/ml; List Biological Laboratories, NC9393111) or vehicle (glycerol) was added to the culture medium, which was changed daily. After 4 days in culture, the explants were fixed either in 4% PFA (Fig. 6) or in TCA for 10 min on ice (Figs 7 and 8) and then gently detached from the dish and transferred to microtubes. Blocking, staining and mounting were performed as described in the ‘Immunolabeling’ section.

### Image acquisition and quantification

Fluorescent images for Figs 1–3 were captured by structured illumination microscopy using a Zeiss Axio Imager M.2 with ApoTome.2 attachments and an Axiocam 506 monochrome camera. Images in Figs 4–8 were captured with a LSM800 line scanning confocal microscope, a 63× NA 1.4 oil objective, the Airyscan detector in confocal mode and Zen 2.3 or Zen 2.6 software (Carl Zeiss AG). To measure HC orientation in cochlear explants (Figs 6–8), images were captured with a Leica DM5500B wide-field microscope, a 63× oil objective, a Hamamatsu ORCA-Flash4.0 sCMOS camera and the Leica Application Suite (LasX) software (Leica Microsystems). All quantifications include at least three animals per genotype and per condition. Animal cohort sizes (N) as well as the number of HC analyzed (n) are indicated on the graphs or in the figure legend.

To measure HC orientation in cochlear wholemounts (Figs 1–3), the tissue was immunolabeled for β2-Spectrin and F-actin. HC orientation was determined using the angle measurement tool in Fiji (NIH) to measure the angle formed by a line drawn from the position of the fonticulus across the center of the HC surface and a second line drawn parallel to the hair cell row along the cochlea longitudinal axis. The fonticulus lacking F-actin or β2-Spectrin signals denotes the position of the basal body. The same approach was used to measure HC orientation in Figs 6–8 using a PCNT antibody to label the basal body and MYO7A or β2-spectrin to label HCs. For freshly harvested tissue in Figs 5–8, 1-3 fields per cochlea and per cochlear position (base, mid and apex) were quantified for each animal (67.61×67.61 μm in Fig. 5; 12.6×12.6 μm in Figs 6–8). For cultured explants at 4 DIV, the entire explant was imaged and orientation measured for all HCs (Figs 6–8). When a result was not quantified, representative images reflect an outcome observed in at least three samples from three animals from two or more litters for each genotype or condition.

To measure GPR156 crescents angle and span in Fig. 5B, OHCs were identified in Fiji by drawing circular ROIs around the ZO1 staining to define cell boundaries. GPR156 crescents were isolated by applying an intensity threshold (35-255) to the GPR156 channel, and crescent ROIs were selected using the magic wand tool. The center of mass for both the cell ROI and the crescent ROI was determined using a custom ImageJ macro that recorded centroid coordinates for each ROI. A reference line parallel to the cochlear longitudinal axis was defined and the angle between this reference line and the vector connecting the cell centroid to the crescent centroid was measured automatically.

To measure GPR156 crescent orientation in Fig. 5E, *z*-stack series were acquired at each cochlear position. A single *z* slice in the stack was chosen based on the strongest GPR156 signal at the junctional level. That signal was then masked using the thresholding tool in Fiji (threshold 35-255) to define the outline of the crescent. Using the angle tool in Fiji, we then measured the angle formed by a line drawn from the center of the GPR156 crescent across the center of the HC surface and a second line parallel to the cochlea longitudinal axis. All angles were measured so that 0° pointed toward the cochlear base and 90° towards the cochlear periphery (lateral).

### Transfection, immunoprecipitation and western blot

HEK293T/17 cells were obtained from the American Type Culture Collection (ATCC) and cultured at 37°C and 5% CO_2_ in DMEM (Gibco, 10567-014) supplemented with 10% fetal bovine serum (FBS; Biowest, S1520), MEM non-essential amino acids (Gibco, 11140-050), sodium pyruvate (Gibco, 11360-070), GlutaMAX (Gibco, 35050-061), and antibiotics (100 units/ml penicillin and 100μg/ml streptomycin; Gibco, 15140-122). Cells were routinely monitored for mycoplasma contamination. 2×10^6^ cells/well were seeded in six-well plates in 1.5 ml of antibiotic-free medium. Cells were transfected after 4 h using linear 25 kDa polyethylenimine (PEI) (Polysciences; 23966) at a 1:3 ratio between total μg of DNA plasmid (2.5 μg) and μl of PEI (7.5 μl, 1 mg/ml). 1.25 μg of a plasmid encoding human GPR156 in frame with an ALFA-tag sequence (SRLEEELRRRLTE) in its N-terminus, preceded by a signal peptide sequence, were co-transfected with 1.25 μg of a plasmid encoding myc-hVANGL1 or myc-hVANGL2. The codon-optimized sequence encoding GPR156 was subcloned from the plasmid in the PRESTO-tango kit (a kind gift from Dr Bryan Roth, University of North Carolina, Chapel Hill, NC, USA; Addgene #66330). Gene fragments encoding the codon-optimized sequences for human VANGL1 (NM_001172412.2) and human VANGL2 (NM_020335.3) with an N-terminal myc tag (M-EQKLISEEDL) were purchased from Twist Bioscience and subcloned into a pcDNA3.1 expression vector. Empty vector pcDNA3.1 was used to normalize the amount of transfected DNA. All the constructs were verified by Sanger sequencing. 24 h after transfection, cells were harvested and lysed in ice-cold immunoprecipitation buffer [300 mm NaCl, 50 mm Tris-HCl (pH 7.4), 1% Triton X-100 and complete protease inhibitor mixture] by sonication. Cell lysates were cleared by centrifugation at 14,000 ***g*** for 15 min, and the supernatants were incubated with 10 μl of ALFA-selector ST magnetic agarose beads (NanoTag Biotechnologies) on a rocker at 4°C for 1 h. After three washes with immunoprecipitation buffer, proteins were eluted with 50 μl of 2× SDS sample buffer and analyzed by SDS-PAGE using a recombinant anti-ALFA Nb Nluc (Porter et al., 2025) or mouse anti-myc antibodies (GenScript, A00704).

### Data plotting and statistics to compare angle distributions

Circular plots of angle frequency distributions were plotted using either the Oriana circular graphing software (Kovach Computing Services) (Figs 1–3) or R (4.2.2) and Rstudio (2022.12.0+353) (Figs 6–8). Circular plots in Figs 6–8 were produced using the R package dyplr and the coord_polar function of the ggplot2 package to organize data and generate the graphs, respectively. The colstats function of the R circular package calculated the circular mean and the mean circular deviation, which are represented by a red line and the length of the arc respectively. In Fig. 5B, data were analyzed and visualized in R. Angles were converted from polar to Cartesian coordinates using trigonometric transformations, and circular plots were generated using ggplot2, where angular position represents GPR156 crescent orientation relative to the longitudinal axis and radial distance corresponds to crescent area. GPR156 and basal body angles in Fig. 5E were plotted in Prism 9 (GraphPad). To compare the angular distribution of HC orientation across stages specifically (Figs 1-3 and 6-8), we used a Watson U^2^ test in R (watson.two.test in CircStats package) with a significance level (α) of 0.001. With a combined sample size of *n*≥18, a U^2^ value>0.385 rejects the null hypothesis that the two distributions are similar, and larger U^2^ values indicate greater dissimilarity between distributions.

## Supplementary Material


