# Supplementary material for: Progressive correction of auditory hair cell orientation in the absence of core planar cell polarity and GPR156 signaling
Source: Development. 2026 May 14;153(9):dev205332. doi: 10.1242/dev.205332 (PMC13245905; doi:10.1242/dev.205332)
Supplement: Supplementary file 1 [file develop-153-205332_review_history.pdf]

Progressive correction of auditory hair cell orientation in the absence of core planar cell polarity and GPR156 signaling

Amandine Jarysta, Justin Nemelka, Muthumeena Ramanathan, Ellison J. Goodrich, Brianna L. Vonderhaar, Sungjin Park, Cesare Orlandi, Michael R. Deans and Basile Tarchini
DOI: 10.1242/dev.205332

Editor: Liz Robertson

Review timeline

Original submission:	27 October 2025
Editorial decision:	28 November 2025
First revision received:	30 March 2026
Accepted:	14 April 2026

Original submission

First decision letter

MS ID#: dev.205332

MS TITLE: Progressive correction of auditory hair cell orientation in the absence of core planar cell polarity and GPR156 signaling

AUTHORS: Amandine Jarysta, Justin Nemelka, Ellison J. Goodrich, Brianna L. Vonderhaar, Sungjin Park, Cesare Orlandi, Michael R. Deans and Basile Tarchini

Dear Dr Tarchini,

I have now received all the referees' reports on the above manuscript, and have reached a decision. The referees' comments are appended below.

As you will see, the referees express considerable interest in your work, but have some significant criticisms and recommend a substantial revision of your manuscript before we can consider publication. If you are able to revise the manuscript along the lines suggested, which may involve further experiments, I will be happy receive a revised version of the manuscript. Your revised paper will be re-reviewed by one or more of the original referees, and acceptance of your manuscript will depend on your addressing satisfactorily the reviewers' major concerns. Please also note that Development will normally permit only one round of major revision. If it would be helpful, you are welcome to contact us to discuss your revision in greater detail. Please send us a point-by-point response indicating your plans for addressing the referees' comments, and we will look over this and provide further guidance.

Please attend to all of the reviewers' comments and ensure that you upload both a 'clean' version of your Word file, along with a highlighted version clearly showing where you have made changes in the revised manuscript. Please avoid using 'Tracked changes' in Word files as these are lost in PDF conversion. I should be grateful if you would also provide a point-by-point response detailing how you have dealt with the points raised by the reviewers in the 'Response to Reviewers' box. If you do not agree with any of their criticisms or suggestions please explain clearly why this is so.

Reviewer 1

© 2026. Published by The Company of Biologists under the terms of the Creative Commons Attribution License (https://creativecommons.org/licenses/by/4.0/).

Advance summary and potential significance to field

In this paper, Jarysta and colleagues investigate the nature of cochlear hair cell reorientation in planar cell polarity mutants. A postnatal correction of misaligned stereocilia bundles was previously reported in Vangl2 KOs, but whether this was due to functional redundancy with other PCP components or a separate mechanism operating in parallel was not understood. This study provides solid evidence that postnatal reorientation is not due to a Vangl1 or Fzd3/6 mechanism, as hair cells still reorient in double conditional knockouts by postnatal day 5. Intriguingly the authors rule out two possible other mechanisms, GPR156 signaling and physical connection with the tectorial membrane. Moreover, the reorientation mechanism appears to be intrinsic to the tissue as it still occurs in cochlear explants grown ex vivo. Postnatal correction of planar polarity misalignment is also observed in mouse hair follicles, and probably in Drosophila wing hair as well (the mutant patterns show local alignment), and thus deciphering this alternative mechanism has broader implications for our understanding of the robustness underlying the formation of highly aligned body structures. Although three solid hypotheses are clearly and convincingly ruled out by the experiments in this study, the mechanism that reorients misaligned hair cells remains unknown.

Comments for the author

1. The authors use different Cre lines to remove Fzd3/6 and Vangl1/2 function conditionally in the cochlea. This is not explained. Do these drivers target the same cells in the ear?
2. In the mouse skin, Fz6 and Vangl2 mutant hair follicles rotate in opposite directions during their postnatal correction (Cetera et al 2017; this work should also be included in the discussion line 346-7). Can the authors tell which direction hair bundles rotate and might the direction be different in different mutants?
3. Since hair bundles correct their orientations postnatally in PCP mutants, does this mean their hearing is normal? If experiments are not feasible, can the authors at least discuss this in the context of what has been previously reported about hearing in PCP mutants.
4. In Fig 1A, I found it difficult to understand how HC orientations were scored at E17.5. The V-shaped stereocilia structure is less defined at this stage and their orientations were hard to appreciate from the images provided.
5. The inclusion of some data regarding the cellular mechanism by which HCs reorient would greatly strengthen the paper and increase the impact. For example, are the cytoskeletal structures alone rotating within the cytoplasm? Or is the entire HC rotating by swapping neighbors through junctional rearrangements? What other morphological changes occur during P0 and P5 that might account for the reorientation?
6. The localization of GPR156 on the Fzd3/6 side of the HC seems at odds with GPR156 dependence on Vangl1/2 for its membrane enrichment and its ability to bind Vangl1/2 in HEK cells. Although the authors do address in this in the discussion, it could be worthwhile to revisit Vangl1/2 localization patterns by super-resolution imaging.
7. Radial histograms require some circular statistics to test for effect size and significant differences between genotypes.

Reviewer 2

Advance summary and potential significance to field

This paper investigated the involvement of the core PCP and Gpr156-Gi pathways in aligning hair bundle orientation in postnatal mouse cochlea. Previous studies using null mutations indicated a role of PCP signaling and Gpr156-Gαi signaling in the embryonic establishment phase of hair cell planar polarity. Using viable conditional knockouts of Vangl1/2 and Fzd3/6, the authors showed that interestingly, hair bundles in postnatal core PCP mutants corrected their misorientation, suggesting of a role of Gpr156-Gαi in the correction process. The found that Vangl1/2 form a complex with and are required for the junctional localization of Gpr156. However, inhibition of Gαi by PTX treatment ex vivo unexpectedly improved hair bundle orientation in core PCP mutant explants, revealing a negative rather than positive role of Gi signaling in the correction process. Overall, the data are extensive, clearly presented and largely convincing. They reveal a

© 2026. Published by The Company of Biologists under the terms of the Creative Commons Attribution License (https://creativecommons.org/licenses/by/4.0/).

compensatory mechanism involving G protein signaling in the postnatal cochlea to align hair cell PCP, although the underlying mechanisms remain to be determined.

Comments for the author

Specific comments:
1. When comparing hair bundle angle measurements between treatments / genotypes / stages (in Figures 1, 2, 3, 6, 7), it would be more convincing to apply appropriate circular statistics (e.g. the Watson's U2 test) to evaluate the statistical significance of the observed differences in hair bundle orientation.
2. Figure 5A. In Fzd3/6 cKO, Gpr156 appeared to form misoriented "half-crescents" in OHCs at the base and accumulate intracellularly in hair cells at the apex, suggesting that Fzd3/6 may influence Gpr156 trafficking to the cell surface, albeit to a lesser extent than Vangl1/2. The results would be more robust with quantifications, and the significance of these results is also worth discussing.
3. Related to above, it was assumed but not shown whether junctional Vangl1/2 was lost in Fzd3/6 cKO ears. Given that asymmetric Gpr156 localization was still observed, it would be important to examine if Vangl1/2 and Gpr156 co-localize in Fzd3/6 cKO hair cells. A positive result would lend support to the idea that, in the absence of Fzd3/6 antagonism, Vangl1/2 can act in hair cells to promote Gpr156 localization, through their proposed cis-interaction.
4. Lines 287-334 and Discussion. The authors equated PTX treatment with inhibiting Gpr156-GNAI signaling. However, PTX is known to inhibit both Gi and Go. Given this caveat and in the absence of genetic evidence, one can only conclude from the ex vivo experiments that PTX-sensitive G proteins play a negative role in postnatal hair bundel reorientation in PCP mutants.
5. Additionally, given the asymmetric localization of Fzd3/6 and Gpr156, it is conceivable that localized Gpr156-Gαi signaling is important for hair cell PCP, whereas PTX will ubiquitously inhibit Gαi and likely have pleiotropic effects beyond Gpr156 signaling.
6. Figures 7, 8. It would be helpful to show representative IF panels of explants with or without PTX treatment, at least from one cochlear region where PTX improved the correction process (e.g. mid).
7. Hair bundle reorientation in core PCP mutants is likely mediated by one of the hair cell-intrinsic planar polarity proteins, most notably the non-receptor GEF Daple and the microtubule motor cytoplasmic dynein. Their potential roles should be discussed, if not experimentally tested.
8. Related to above, non-canonical Wnt signaling may also be involved in postnatal hair bundle reorientation, for example, by regulating Gi signaling through the non-receptor GEF Daple (PMID: 12702652; PMID: 39172791). This possibility should also be discussed.
9. Line 435. "(FZD>VANGL or FZD>VANGL)" seems to be a typo.
10. Can the authors comment on whether the cochlear duct length was decreased in Vangl1/2 and Fzd3/6 cKO mutants? Past studies of PCP null mutants suggested that PCP signaling controls convergent extension movements during cochlear duct extension. However, it is possible that shortened cochlear ducts in PCP null mutants is due to secondary effect of neural tube defect. the CKO mutants would present an opportunity to distinguish between these possibilities.

First revision

Author response to reviewers' comments

We would like to thank the Reviewers for their time and thoughtfoul comments. We have revised the manuscript to incorporate several changes as detailed below point by point.

Reviewer 1: SUMMARY OF THE ADVANCE MADE IN THIS PAPER AND ITS POTENTIAL SIGNIFICANCE TO THE FIELD

In this paper, Jarysta and colleagues investigate the nature of cochlear hair cell reorientation in planar cell polarity mutants. A postnatal correction of misaligned stereocilia bundles was previously reported in Vangl2 KOs, but whether this was due to functional redundancy with other PCP components or a separate mechanism operating in parallel was not understood. This study

© 2026. Published by The Company of Biologists under the terms of the Creative Commons Attribution License (https://creativecommons.org/licenses/by/4.0/).

provides solid evidence that postnatal reorientation is not due to a Vangl1 or Fzd3/6 mechanism, as hair cells still reorient in double conditional knockouts by postnatal day 5. Intriguingly the authors rule out two possible other mechanisms, GPR156 signaling and physical connection with the tectorial membrane. Moreover, the reorientation mechanism appears to be intrinsic to the tissue as it still occurs in cochlear explants grown ex vivo. Postnatal correction of planar polarity misalignment is also observed in mouse hair follicles, and probably in Drosophila wing hair as well (the mutant patterns show local alignment), and thus deciphering this alternative mechanism has broader implications for our understanding of the robustness underlying the formation of highly aligned body structures. Although three solid hypotheses are clearly and convincingly ruled out by the experiments in this study, the mechanism that reorients misaligned hair cells remains unknown.

SUGGESTIONS TO AUTHORS

1. The authors use different Cre lines to remove Fzd3/6 and Vangl1/2 function conditionally in the cochlea. This is not explained. Do these drivers target the same cells in the ear?

The rationale for using Pax2-Cre to generate Vangl1/2 CKOs and Slc26a9-Cre to generate Fzd3/6 CKOs did not reflect differences in experimental design but rather implementation. At the time the Vangl1/2 experiments were conducted Pax2-Cre was the only reliable driver for generating whole ear CKOs. When Slc26a9-Cre became available we switched to this line because it was more specific to the ear with less expression in other embryonic tissues. The Fzd3/Fzd6 analysis occurred after this switch. A few Fzd3/6 CKOs were also generated using an Emx2-Cre driver and were combined with Slc26a9-Cre generated CKOs (Fig.3) since identical phenotypes were seen at P0 for both. This is noted in the legend and methods section.

Importantly, all three Cre drivers meet criteria necessary for this study: (1) Expression that starts at early stages of inner ear development and prior to hair cell specification, (2) Expression throughout the cochlear duct and in both hair cells and supporting cells, and (3) no expression in the developing neural tube which prevented lethal neural tube defects.

We have added a clear description of the spatial and temporal patterns of recombination and corresponding references for these drivers where they first appear in the manuscript.

2. In the mouse skin, Fz6 and Vangl2 mutant hair follicles rotate in opposite directions during their postnatal correction (Cetera et al 2017; this work should also be included in the discussion line 346-7). Can the authors tell which direction hair bundles rotate and might the direction be different in different mutants?

Studies in this manuscript exclusively involve fixed samples, so we are unable to define how hair cells achieve the orientation they exhibit at tissue harvesting time. However, our data do not suggest a bias in the direction of a possible rotation (e.g., more angles in the 90°-270° range if mutant hair cells pointing medially (270°) were mostly rotating clockwise to adopt a normal lateral (90°) orientation).

Strictly speaking, we actually do not know that hair cells are rotating. Alternatively, only the apical cytoskeleton could be remodeled at the apical membrane. New studies involving live-imaging are underway to address these important questions and will be reported in a separate manuscript.

We added the Cetera 2017 reference to the discussion, as well as the notion that Vangl2 mutants, like Fzd6 mutants, are able to correct hair follicle orientation. Thank you for pointing out this omission.

3. Since hair bundles correct their orientations postnatally in PCP mutants, does this mean their hearing is normal? If experiments are not feasible, can the authors at least discuss this in the context of what has been previously reported about hearing in PCP mutants.

This question was addressed previously in Copley et al., 2013 in a conditional Vangl2 model. In these mice auditory defects were detected, however the study could not resolve whether this was

© 2026. Published by The Company of Biologists under the terms of the Creative Commons Attribution License (https://creativecommons.org/licenses/by/4.0/).

due to hair cells at the cochlear apex that did not fully correct their orientation, or structural changes to the supporting cells surrounding hair cells that might impact vibration. Both could contribute to hearing impairment.

This background information was not mentioned in the original manuscript and has now been added to the Introduction (line 111) along with mention of Gpr156 hearing loss for completeness.

In this new work that involves multiple core PCP models and interrogates the role of GPR156-GNAI, we are not testing hearing because we expect similar cellular defects and functional deficits as in the *Vangl2* model. In addition, it was not realistic to perform ABR on Vangl1;2 and Fzd3;6 double conditional mutants given the low odds and reduced fitness of these animals.

4. In Fig 1A, I found it difficult to understand how HC orientations were scored at E17.5. The V-shaped stereocilia structure is less defined at this stage and their orientations were hard to appreciate from the images provided.

At E17.5 we rely less on the shape of the hair bundle than on the position of the off-center basal body indicated by the fonticulus (the F-actin gap in the cuticular plate at the position of the basal body).

This was explained previously in Results (now Line 152) and in Methods (now Line 597). However, we recognize that this could have been made clearer and modified Figure 1A by adding an image inset to clarify this point and a reference system for angle measurements.

5. The inclusion of some data regarding the cellular mechanism by which HCs reorient would greatly strengthen the paper and increase the impact. For example, are the cytoskeletal structures alone rotating within the cytoplasm? Or is the entire HC rotating by swapping neighbors through junctional rearrangements? What other morphological changes occur during P0 and P5 that might account for the reorientation?

The cellular modality behind reorientation remains obscure. This could be an actual rotation of the whole hair cell, a rotation of the intact apical cytoskeleton at apical junction, or a more complex remodeling where different cytoskeletal elements (stereocilia, basal body, cuticular plate, microtubules) behave more independently.

Resolving this question will require high magnification/resolution live imaging of explanted cochlea at late embryonic stages. Such experiments are currently underway but fall outside the scope of the present study.

The auditory epithelium is evolving past birth as, for example, supporting cells modify their baso-lateral morphology to adopt specialized 'phalangeal' processes and a more elongated cell body. However, supporting cell-hair cell contacts at apical junctions are not fundamentally changed past birth, and do not provide an obvious testable hypothesis for the reorientation modality.

6. The localization of GPR156 on the Fzd3/6 side of the HC seems at odds with GPR156 dependence on Vangl1/2 for its membrane enrichment and its ability to bind Vangl1/2 in HEK cells. Although the authors do address in this in the discussion, it could be worthwhile to revisit Vangl1/2 localization patterns by super-resolution imaging.

Vangl2 localization was previously addressed with STED immunolabeling in Giese et al., 2012. This study is cited and concluded that Vangl2 is primarily on the supporting cell side, but did not rule out protein enrichment on the hair cell side, noting that Vangl2 is expressed in both cell types. This was not quantified and we would argue that the results were more suggestive than definitive.

As an alternative approach to revisit protein localization at the hair cell- support cell junction, we exploited a recently developed tdTomato-Vangl2 knock-in mouse model (Basta et al., 2021). Interestingly, when fixing tissue with TCA as in Giese et al., we observed intracellular tdTomato signals on both sides of the loosened apical junction in approximatively equal amounts. This is now shown in Figure 4C and brought up after establishing GPR156-VANGL2 interaction in HEK293

© 2026. Published by The Company of Biologists under the terms of the Creative Commons Attribution License (https://creativecommons.org/licenses/by/4.0/).

cells. This result supports that VANGL2 protein is also present on the medial hair cell side, adjacent to GPR156, and may thus interact with GPR156 in hair cells as in HEK293 cells. Text in Results was modified accordingly line 241.

7. Radial histograms require some circular statistics to test for effect size and significant differences between genotypes.

Reviewer 2 had a similar request and mentioned Watson U^2 as a possible test. We have applied this test to specifically compare stages in control and mutant conditions in Figures 1-3 and 6-8, as indicated (U^2 value added as caption). A U^2 value > 0.385 rejects the null hypothesis that two distributions are similar, and larger U^2 values indicate greater dissimilarity between distributions.

Reviewer 2: SUMMARY OF THE ADVANCE MADE IN THIS PAPER AND ITS POTENTIAL SIGNIFICANCE TO THE FIELD

This paper investigated the involvement of the core PCP and Gpr156-Gi pathways in aligning hair bundle orientation in postnatal mouse cochlea. Previous studies using null mutations indicated a role of PCP signaling and Gpr156-Gai signaling in the embryonic establishment phase of hair cell planar polarity. Using viable conditional knockouts of Vangl1/2 and Fzd3/6, the authors showed that interestingly, hair bundles in postnatal core PCP mutants corrected their misorientation, suggesting of a role of Gpr156-Gai in the correction process. The found that Vangl1/2 form a complex with and are required for the junctional localization of Gpr156. However, inhibition of Gai by PTX treatment ex vivo unexpectedly improved hair bundle orientation in core PCP mutant explants, revealing a negative rather than positive role of Gi signaling in the correction process. Overall, the data are extensive, clearly presented and largely convincing. They reveal a compensatory mechanism involving G protein signaling in the postnatal cochlea to align hair cell PCP, although the underlying mechanisms remain to be determined.

SUGGESTIONS TO AUTHORS

Specific comments:
1. When comparing hair bundle angle measurements between treatments / genotypes / stages (in Figures 1, 2, 3, 6, 7), it would be more convincing to apply appropriate circular statistics (e.g. the Watson's U2 test) to evaluate the statistical significance of the observed differences in hair bundle orientation.

Please see reply above for point 7 raised by Reviewer 1.

2. Figure 5A. In Fzd3/6 cKO, Gpr156 appeared to form misoriented "half-crescents" in OHCs at the base and accumulate intracellularly in hair cells at the apex, suggesting that Fzd3/6 may influence Gpr156 trafficking to the cell surface, albeit to a lesser extent than Vangl1/2. The results would be more robust with quantifications, and the significance of these results is also worth discussing.

Half-crescents are indeed observed in OHCs and we previously failed to point this out. Text in Results has been modified accordingly, and we now quantify this phenomenon in Figure 5B. As this is observed at the cochlear base only (P0), we conclude that this is a maintenance issue where GPR156 is no longer able to be deployed along the entire medial half of the apical junction over time in absence of FZD3/6. The main text has been modified accordingly starting Line 256.

As for the "intracellular accumulation" at the apex, this is unspecific labeling by the GPR156 antibody which was visible on some samples and not others. We swapped that mutant panel that now looks similar to the apex control panel except for the inverted (lateral) crescents in IHCs. Please note that as stated in the text already, the GPR156 antibody also unspecifically labeled the basal body region in both controls and mutants. This is not usually observed with this antibody and we suspect that delays associated with shipping TCA-fixed samples from Utah to Maine for immunolabeling played a role.

3. Related to above, it was assumed but not shown whether junctional Vangl1/2 was lost in Fzd3/6 cKO ears. Given that asymmetric Gpr156 localization was still observed, it would be important to

© 2026. Published by The Company of Biologists under the terms of the Creative Commons Attribution License (https://creativecommons.org/licenses/by/4.0/).

examine if Vangl1/2 and Gpr156 co-localize in Fzd3/6 cKO hair cells. A positive result would lend support to the idea that, in the absence of Fzd3/6 antagonism, Vangl1/2 can act in hair cells to promote Gpr156 localization, through their proposed cis- interaction.

The fate of VANGL1/2 in *Fzd3/Fzd6* mutants has not been previously reported specifically, although the converse was (loss of FZD protein in Vangl2 Looptail mutants; Wang et al., 2006, Montcouquiol et al., 2006). Consequently, to verify the general expectation of inter- dependence amongst core PCP members, we attempted to examine the relative distributions of VANGL2 and GPR156 in *Fzd3/Fzd6* DKO. Unfortunately, the resulting changes in VANGL2 distribution are not compatible with the recommended colocalization experiment (see figure below).

When evaluated at P0 in the absence of FZD3 and FZD6, the polarized distribution of VANGL2 is lost. Curiously this occurs differently at junctions with the two types of hair cell. First, at the junctions surrounding OHCs, the polarized crescents of VANGL2 are significantly reduced if not lost altogether (examples indicated by yellow arrows). Second, at the junctions of IHCs, VANGL2 is still present but its distribution is changed so that it is no longer polarized and instead is evenly distributed around the periphery of supporting cells (examples indicated by yellow arrowheads). We presume that there is a corresponding redistribution of VANGL2 in IHCs. However, in these experiments it is not possible to distinguish IHC-derived and supporting cell-derived VANGL2. As a result, we cannot confidently evaluate the colocalization of GPR156 and VANGL2 within IHCs of the *Fzd3/Fzd3* CKO as suggested.

NOTE: We have removed unpublished data that had been provided for the referees in confidence.

4. Lines 287-334 and Discussion. The authors equated PTX treatment with inhibiting Gpr156-GNAI signaling. However, PTX is known to inhibit both Gi and Go. Given this caveat and in the absence of genetic evidence, one can only conclude from the ex vivo experiments that PTX-sensitive G proteins play a negative role in postnatal hair bundel reorientation in PCP mutants.

To clarify, there is solid evidence that GPR156 specifically signals through inhibitory G proteins (Gi/o) to orient hair cells, and that ptx fully recapitulates hair cell misorientation observed in Gpr156 inactivation in both the cochlea and vestibular system. Ptx can thus reliably be used to block GPR156-GNAI signaling. Most relevant to this study, expressing ptx catalytic subunit (ptxA) closely mirrors Gpr156 defects in the auditory epithelium with inverted OHC1-2 and more normal orientation in OHC3 and IHC (Kindt et al. 2021). This unique misorientation signature has not been reported in any other mutant context. As expected, ptxA recapitulates Gpr156 phenotypes without altering GPR156 distribution at the plasma membrane since ptxA targets the signaling pathway downstream of the GPR156 receptor. In addition, Gpr156 was demonstrated to signal via Gai in cell culture assays (Watkins and Orlandi, 2021; PMID 33784795). In this manuscript, we directly verify that ptx holoenzyme can be used to specifically block Gpr156 activity by assessing OHC1-2 orientation post-culture in each experiment (Fig 7A, Fig 8A): OHC1 are inverted as expected, and we thus conclude that orientation outcomes in IHC and OHC3 reflect the elusive correction process ex vivo compounded with blocking Gpr156 activity.

Go is not required for normal hair cell orientation (PCP) or cytoskeleton polarization (intrinsic planar polarity). This was established in Jarysta et al., 2024 (PMID 38651641) which includes Go mutant mice models. Consequently, Go inactivation by ptx is not expected to impact hair cell orientation in core PCP mutants, including the correction process.

Ptx has other effects on hair cells but these do not influence orientation per se, please see response to point 5 below.

5. Additionally, given the asymmetric localization of Fzd3/6 and Gpr156, it is conceivable that localized GPCR-Gαi signaling is important for hair cell PCP, whereas PTX will ubiquitously inhibit Gαi and likely have pleiotropic effects beyond Gpr156 signaling.

Ptx will ribosylate and impair Gai function downstream of GPR156 at the medial hair cell junction. We know that medially-polarized GPR156-Gai signaling is critical for proper hair cell orientation, as for example Stk32a inactivation prevents proper GPR156 polarization, resulting in variable orientation defects (Jia et al., 2023; PMID 37144879 and Jia et al., 2025; PMID

© 2026. Published by The Company of Biologists under the terms of the Creative Commons Attribution License (https://creativecommons.org/licenses/by/4.0/).

41208475).

It is correct that ptx also affects the GPSM2-Gai complex, but in contrast to core PCP and GPR156, GPSM2-Gai is not involved in regulating hair cell orientation per se and does not include a transmembrane protein at the apical cell junction. Instead, GPSM2-Gai is active at the apical membrane and regulates cytoskeleton polarization (i.e. intrinsic planar polarity). Inactivating GPMS2-Gai genetically or via ptx generate similar phenotypes: variably misplaced and severely shortened stereocilia in the hair bundle, defects that are most obvious at postnatal stages, well after orientation is first established. It is thus unlikely that GPSM2-Gai inactivation by PTX would facilitate correction in this new study.

Of note, reciprocally, GPR156-Gai does not influence intrinsic cytoskeleton polarization (Kindt et al., 2021).

6. Figures 7, 8. It would be helpful to show representative IF panels of explants with or without PTX treatment, at least from one cochlear region where PTX improved the correction process (e.g. mid).

We added representative illustrations in both figures. In Fig. 7C, ptx does not obviously improve orientation at the cochlear mid so we selected an apical view for IHC and OHC3. Please note that both cell types are improperly aligned in Vangl2 Lp mutants. In Fig. 8, IHC orientation is improved at all positions and we provide an illustration at the cochlear mid. IHC alignment is relatively normal by contrast in this mutant.

7. Hair bundle reorientation in core PCP mutants is likely mediated by one of the hair cell-intrinsic planar polarity proteins, most notably the non-receptor GEF Daple and the microtubule motor cytoplasmic dynein. Their potential roles should be discussed, if not experimentally tested.

Daple was proposed to spatially coordinate PCP (orientation) with intrinsically polarized cytoskeleton based on its known molecular roles and mutant defects, which span both levels of planar polarity (i.e. dysmorphic <u>and</u> severely misoriented hair bundles). It is arguable whether DAPLE, or other proteins proposed to couple PCP and the cytoskeleton, are prime candidates for the correction process. In core PCP and *Gpr156* mutants, the cytoskeleton remains largely intact throughout all stages, and this might instead indicate that correction involves other factors specifically regulating hair cell orientation at cell-cell junctions. This remains entirely speculative, and please note that testing the role of Daple for example would be challenging as it would require breeding Daple with conditional Vangl or Fzd mutants. See also reply to the next point 8.

8. Related to above, non-canonical Wnt signaling may also be involved in postnatal hair bundle reorientation, for example, by regulating Gi signaling through the non-receptor GEF Daple (PMID: 12702652; PMID: 39172791). This possibility should also be discussed.

Points 7 and 8 were taken into account and the Discussion was edited to add a mention of other Gai-related protein and pathways besides Fat-Dachsous (Line 401).

9. Line 435. "(FZD>VANGL or FZD>VANGL)" seems to be a typo.

Yes, and this was corrected to read FZD>VANGL or VANGL>FZD.

10. Can the authors comment on whether the cochlear duct length was decreased in Vangl1/2 and Fzd3/6 cKO mutants? Past studies of PCP null mutants suggested that PCP signaling controls convergent extension movements during cochlear duct extension. However, it is possible that shortened cochlear ducts in PCP null mutants is due to secondary effect of neural tube defect. the CKO mutants would present an opportunity to distinguish between these possibilities.

We agree that this is an interesting consideration for PCP mutants and note that the comparison has already been made for Vangl2. Copley et al. 2013 compared Pax2-Cre, Vangl2 CKO cochlea to Vangl2 KOs with neural tube defects and showed that the KO cochlea was

© 2026. Published by The Company of Biologists under the terms of the Creative Commons Attribution License (https://creativecommons.org/licenses/by/4.0/).

shorter than the CKO and littermate controls (which were comparable to each other). Although we have not measured cochlear length for the Vangl1/Vangl2 or Fzd3/Fzd6 double mutants there is no indication that they are shorter than controls and we never saw extra rows of OHCs at the cochlear apex that would indicate defects in convergent extension. We now cite this previous result in the Discussion, and briefly indicate that cochlear length does not appear to be affected in double Vangl or Fzd mutants either (Line 404).

Second decision letter

MS ID#: dev.205332R1

MS TITLE: Progressive correction of auditory hair cell orientation in the absence of core planar cell polarity and GPR156 signaling

AUTHORS: Amandine Jarysta, Justin Nemelka, Muthumeena Ramanathan, Ellison J. Goodrich, Brianna L. Vonderhaar, Sungjin Park, Cesare Orlandi, Michael R. Deans and Basile Tarchini

Dear Dr Tarchini,

I am happy to tell you that your manuscript has been accepted for publication in Development, pending our standard publication integrity checks.

Reviewer 1

Advance summary and potential significance to field

The authors have nicely addressed the majority of the reviewers comments, and the questions that remain are outside of the scope of the current study. This is a nicely-executed study that comprehensively interrogates the genetic interactions that mediate hair bundle misorientation and correction in the cochlea.

Reviewer 2

Advance summary and potential significance to field

This paper investigated the correction of hair bundle orientation during postnatal development in core PCP mutant mice. Previous studies using null mutations indicated a role of PCP signaling and Gpr156-Gai signaling in the embryonic establishment phase of hair cell PCP. Using viable conditional knockouts of Vangl1/2 and Fzd3/6, the authors showed that hair bundle misorientation was significantly corrected postnatally, suggesting a core-PCP independent mechanism. Using Tecta knockout mice and Ptx inhibition of Gi signaling in cochlear explants, respectively, it was demonstrated that the tectorial membrane and the Gpr156-Gi signaling axis are dispensable for postnatal correction of hair bundle misorientation in core PCP mutants. The study also showed that Vangl1/2 form a complex with Grp156 in cultured cells, and they may interact in cis on hair cell membranes to promote asymmetric junctional localization of Gpr156.
Overall, the data are extensive, clearly presented and convincing, although the hair bundle reorientation mechanisms remain to be determined.

Comments for the author

The authors have addressed all the issues raised in the last submission. There are no further questions.

© 2026. Published by The Company of Biologists under the terms of the Creative Commons Attribution License (https://creativecommons.org/licenses/by/4.0/).
